# SUMOylation of Translationally Regulated Tumor Protein Modulates Its Immune Function

**DOI:** 10.3389/fimmu.2022.807097

**Published:** 2022-02-07

**Authors:** Chenchen Lu, Zhiqing Li, Wenchang Zhang, Hao Guo, Weiqun Lan, Guanwang Shen, Qingyou Xia, Ping Zhao

**Affiliations:** ^1^ State Key Laboratory of Silkworm Genome Biology, Biological Science Research Center, Southwest University, Chongqing, China; ^2^ Chongqing Key Laboratory of Sericultural Science, Chongqing Engineering and Technology Research Center for Novel Silk Materials, Southwest University, Chongqing, China

**Keywords:** SUMOylation, BmNPV, *Bombyx mori*, TCTP, antivirus

## Abstract

Translationally controlled tumor protein (TCTP) is a highly conserved protein possessing numerous biological functions and molecular interactions, ranging from cell growth to immune responses. However, the molecular mechanism by which TCTP regulates immune function is largely unknown. Here, we found that knockdown of *Bombyx mori* translationally controlled tumor protein (*BmTCTP*) led to the increased susceptibility of silkworm cells to virus infection, whereas overexpression of *BmTCTP* significantly decreased the virus replication. We further demonstrated that *BmTCTP* could be modified by SUMOylation molecular BmSMT3 at the lysine 164 *via* the conjugating enzyme BmUBC9, and the stable SUMOylation of *BmTCTP* by expressing *BmTCTP*-BmSMT3 fusion protein exhibited strong antiviral activity, which confirmed that the SUMOylation of *BmTCTP* would contribute to its immune responses. Further work indicated that *BmTCTP* is able to physically interact with interleukin enhancer binding factor (ILF), one immune molecular, involved in antivirus, and also induce the expression of *BmILF* in response to virus infection, which in turn enhanced antiviral activity of *BmTCTP*. Altogether, our present study has provided a novel insight into defending against virus *via BmTCTP* SUMOylation signaling pathway and interacting with key immune molecular in silkworm.

## Introduction

Translationally controlled tumor protein (TCTP), also named *tumor protein translationally controlled 1 (TPT1)*, histamine release factor (HRF), fortilin, *p23*, or Mmi, was originally identified as a protein that undergoes an early and prominent increase upon serum stimulation in murine cell lines (1–4) and thus establishing TCTP as a translationally regulated protein (5). It was subsequently found that TCTP has been an evolutionary conserved protein present in eukaryotic organisms, including mammals, plants, and insects (6–9). TCTP has been characterized as a multifunction protein that participates in multiple biological processes, such as cell growth, cell cycle progression, cell apoptosis, protein synthesis, immune responses, tumor reversion, and tumorigenesis (10–14). Due to the important function in tumorigenesis and upregulated expression in tumors, TCTP has been considered as a promising therapeutic target for cancer prevention and intervention (15, 16).

Given the important roles of TCTP in various species, deciphering the molecular mechanisms underlying TCTP involvement in cellular processes will be critical for understanding its diverse biological functions. One of the important ways is to identify the specific interactions with TCTP, which could provide the potential mechanisms to regulate target processes. Recently, a majority of proteins have been demonstrated to be interacting with TCTP using coimmunoprecipitation (Co-IP) followed by liquid chromatography-tandem mass spectrometry (LC-MS/MS) analysis (17–19). For example, TCTP could interact with cytoskeleton proteins, such as Tubulin and Actin, and participate in regulating microtubule organization and cell morphology (20, 21). TCTP also interacts with ribosomal proteins and translation elongation factors, indicating its role in the translation process (17, 19). By interacting with Bcl-xL and Mcl-1, TCTP increases their stability and promotes antiapoptotic effects (22, 23). TCTP is able to form a complex with p53 and MDM2 and further regulates the degradation of p53 (24–26). The interaction of TCTP with Na,K-ATPase inhibits the pumping activity of Na,K-ATPase and induces Na,K-ATPase-mediated tumorigenic signaling pathways (27, 28).

Posttranslational modification (PTM) of a protein can also determine the target protein activity, localization, and stabilization (29). Another way to understand TCTP function is to investigate its modification processes. It has been demonstrated that human TCTP can be phosphorylated by polo-like kinase, and this phosphorylation inhibits the microtubule-stabilizing activity of TCTP, which in turn regulates the progression of cell cycle (30, 31). Increased phosphorylation of TCTP has also been shown to be associated with a poor clinical response to trastuzumab therapy in human epithelial growth factor receptor-2 (HER2)-positive breast cancer (15). In addition to TCTP phosphorylation, a recent report shows that human TCTP is SUMOylated by small ubiquitin-like modifier (SUMO) at a lysine 164 site. The SUMOylation of TCTP regulates its nuclear transport and promotes antioxidant function of TCTP (32). These modifications on TCTP have shed light on the novel regulation of TCTP activity in organisms.

The demonstrated silkworm *Bombyx mori* is an economically important insect for silk production and an excellent lepidopteran model for studying gene functions (33–35). The role of *Bombyx mori* translationally controlled tumor protein (*BmTCTP*) in the silkworm is emerging in recent years. Clustered regularly interspaced short palindromic repeat/CRISPER-associated protein 9 (CRISPR/Cas9)-mediated depletion of *BmTCTP* led to influenced cell size, cell proliferation, and differentiation of intestinal epithelial cells and thus delayed development and lethality in the third instar larvae of the silkworm (36). Additionally, transgenic RNA interference (RNAi) knockdown of the midgut *BmTCTP* expression suppressed the antimicrobial capacity and the intestinal innate immunity of the silkworm during oral microbial challenge (37, 38). All these results have demonstrated the roles of *BmTCTP* in regulating larval development and antimicrobial activity. However, the molecular mechanism underlying *BmTCTP* involved in the immunity is largely unknown.

To further understand the possible mechanism of *BmTCTP* in the immune response, we here focused on the modification of *BmTCTP* and investigated its role in *Bombyx mori nucleopolyhedrovirus* (BmNPV) infection, one of the serious diseases in sericulture (39, 40). Our present data confirmed that the silkworm *BmTCTP* can be SUMOylated at a conserved lysine site, and the SUMO conjugating enzyme BmUBC9-mediated SUMOylation of *BmTCTP* contributes to its immune response. Furthermore, we showed that *BmTCTP* may act as antiviral immunity *via* regulating the expression of interleukin enhancer binding factor (ILF).

## Materials and Methods

### Plasmids

Full-length cDNAs of *BmTCTP* and *BmILF* were amplified from the cDNA library of cultured silkworm BmN cells by using primers listed in **Table S1** and further cloned into an NcoI–XhoI site of pENTR11 (Invitrogen, Carlsbad, CA, USA) vector. Plasmids expressing *BmTCTP* and *BmILF* with different tags (FLAG, HA, EGFP, and Red) were generated using the gateway technology (Invitrogen, Carlsbad, CA, USA) (41). BmSMT3 and BmUBC9 plasmids were from our previous publication (42). Point mutant of *BmTCTP* at K164 site was generated by PCR-based mutagenesis (43). To tag *BmTCTP* with a BmSMT3 chain, the silkworm *BmSMT3* gene was cloned into the pENTR11 vector, and the resulting plasmid was further ligated with the PCR product of *BmTCTP* (43). All plasmids were verified by sequencing.

### Cell Lines

The silkworm ovary-derived BmN cells were cultured in TC-100 (Sigma, St. Louis, MO, USA) medium supplemented with 10% fetal bovine serum (Hyclone, Logan, UT, USA) and penicillin-streptomycin (Thermo Fisher Scientific, Waltham, MA, USA) at 27°C, respectively.

The expression vectors for FLAG-TCTP and FLAG-TCTP-K164R were inserted into the genome of BmN cells by using piggyBac transposition system according to the previous report, and the stably transformed cells were selected by puromycin (Beyotime, Shanghai, China).

### Sequence Analysis

We performed multiple sequence alignments using CLC sequence viewer 7 based on the silkworm *BmTCTP* and Human HsTCTP. The potential modification of lysine residue was indicated by an asterisk.

### RNA Interference

The synthesis of double-stranded RNAs (dsRNAs) for *BmTCTP* was carried out by T7 RNA polymerase (Promega, Madison, WI, USA) *in vitro* according to the previous report (44). The dsRNAs for control gene of Red were also synthesized. G5-PAMAM-mediated delivery of dsRNA was used according to the previous protocol (45). G5-PAMAM was purchased from Chenyuan Company (Shandong, China).

### PCR

Total RNA samples were prepared from BmN cells using TRIzol reagent (Invitrogen, Carlsbad, CA, USA), and 1 μg of total RNA was used for cDNA synthesis according to the manufacturer’s protocol of the M-MLV Reverse Transcriptase Kit (Promega, Madison, WI, USA). Quantitative real-time polymerase chain reaction (qRT-PCR) was performed with an SYBR Premix ExTaq Kit (Takara, Kyoto, Japan) and a qTower 2.2 Real-time PCR Detection System. The silkworm eukaryotic translation initiation factor 4A (BmeIF-4a), BmActin3, and glyceraldehyde-3-phosphate dehydrogenase (BmGAPDH) genes were used as the internal control. All experiments were independently performed with three biological replicates, and all primers used for PCR were listed in **Table S1**.

### Virus Infection

BmNPV-expressing green fluorescent protein (BmNPV-GFP) was stored in our laboratory of Biological Science Research Center (46). BmN cells infected with BmNPV-GFP at the different time points were collected for different assays.

### Virus Proliferation

BmNPV-GFP-infected BmN cells were harvested at the indicated time points and suspended in phosphate-buffered saline (PBS). Total DNA from each sample was prepared by using a TaKaRa MiniBEST Universal Genomic DNA Extraction Kit (Takara, Kusatsu, Japan) according to the manufacturer’s protocol. The viral DNA abundance of BmNPV was examined by the expression of virus *GP41* gene (46), and the silkworm *GAPDH* gene was used as the internal control (**Table S1**). The viral fluorescence was observed at 72 h post infection (hpi), and the aggregated nuclear DNA was counterstained by 4’,6-diamidino-2-phenylindole (DAPI; Invitrogen, Carlsbad, CA, USA). Fluorescence signals were captured by a fluorescent microscope (Z16, Leica, Wetzlar, Germany).

### Fluorescence Microscopy

For subcellular localization analysis, cells expressing Red-TCTP, Red-TCTP-K164R, Red-TCTP-SMT3, or EGFP-ILF were cultured on a coverslip, fixed with 3.7% formaldehyde in PBS for 10 min, and the nuclear DNA was counterstained by DAPI. Fluorescence signals were captured by a fluorescence microscope (Z16, Leica, Wetzlar, Germany).

### Immunoprecipitation and Immunoblotting

Immunoprecipitation was carried out as described previously (41). Briefly, cells co-expressing proteins were lysed in radio-immunoprecipitation assay (RIPA) buffer (50 mM Tris-HCl, pH 8.0, 150 mM NaCl, 1% Nonidet P-40, 0.5% sodium deoxycholate, 0.1% sodium dodecyl sulfate (SDS)] supplemented with protease inhibitor (Complete, EDTA-free, Roche, New Zealand). The lysates were immunoprecipitated by using an anti-FLAG M2 Affinity Gel (F2426, Sigma, St. Louis, MO, USA), and the eluted proteins were further detected by immunoblotting using anti-HA (AF518, Beyotime, Shanghai, China), anti-FLAG (AH159, Beyotime, Shanghai, China), anti-Smt3 (ab135758, Abcam, Cambridge, UK), and anti-Tubulin (ab7291, Abcam, Cambridge, UK) antibodies.

### Liquid Chromatography-Tandem Mass Spectrometry Assay

After digestion of the immunoprecipitated *BmTCTP* complex, liquid chromatography-tandem mass spectrometry (LC-MS/MS) was carried out to identify the protein interactions according to the previous protocol (47). Briefly, the protein solution after immunoprecipitation was chemically reduced with 10 mM dithiothreitol (DTT) for 1 h at 37°C and then alkylated with 50 mM iodoacetamide for 1 h at room temperature in the dark. After washing with 8 M urea and 50 mM NH_4_HCO_3_ in an ultrafiltration tube, proteins were digested with trypsin for 20 h at 37°C. The peptide mixture was acidified by 0.1% formic acid and resolved by using a Thermo Fisher Scientific EASY-nLC 1000 system (Waltham, MA, USA) under the standard parameters.

### Data Analysis

Protein identification was analyzed with MaxQuant software (version 1.3.0.1) against an integrated silkworm proteome database. The search parameters for protein identification were set according to the published procedure (48). At least one unique peptide was designated as an identified protein, and the protein information was listed in **Table S2**. For the functional annotation of the identified proteins, we used the Blast2GO program (https://www.blast2go.com/) (49) to search against the non-redundant protein database (NR, NCBI, https://www.ncbi.nlm.nih.gov/). The WEGO database (http://wego.genomics.org.cn/) was used to analyze the interacting proteins.

### Statistical Analysis

Statistical data are presented as the mean ± standard deviation (SD) of three independent biological replicates. The significance (P-value) was analyzed by the Student’s t-test and denoted as follows: ^*^P < 0.05, ^**^P <0.01, and ^***^P < 0.001.

## Results

### *BmTCTP* Was SUMOylated at a Conserved Lysine Site in Silkworm

**Figure 1:**
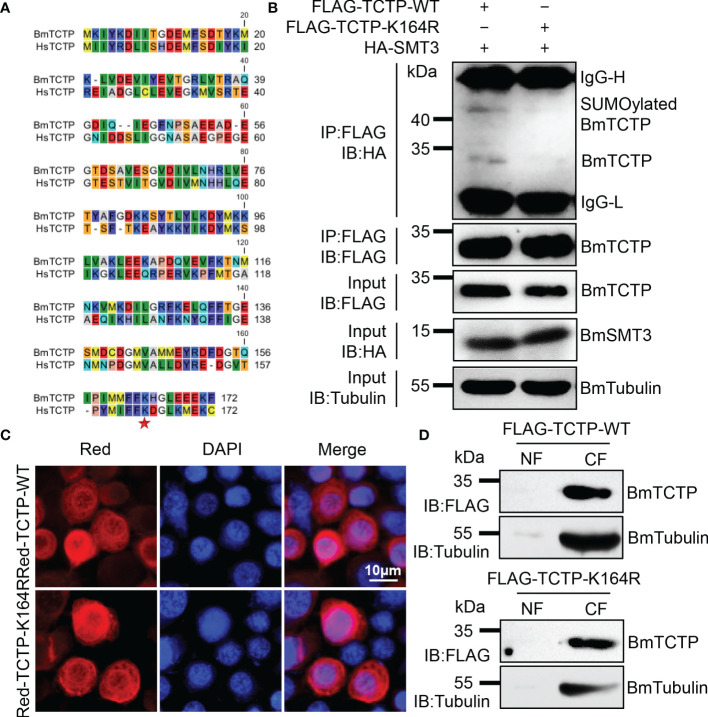
*Bombyx mori* translationally controlled tumor protein (*BmTCTP*) was SUMOylated at a conserved lysine site in silkworm. **(A)** The amino acid sequence alignment was performed using the CLC sequence viewer 7 software on human and silkworm sequences of TCTP. Asterisk indicated residue of the potential SUMOylation site. **(B)** Plasmids as indicated were co-transfected into the silkworm BmN cells. Lysates from BmN cells were used for immunoprecipitation with anti-FLAG antibody, followed by immunoblotting with indicated antibodies. IgG-L and IgG-H were the light and heavy chains of anti-FLAG antibody. **(C)** Subcellular localization of Red-TCTP-WT or Red-TCTP-K164R fusion protein in BmN cells was determined by fluorescence (red), and the nuclear DNA was counterstained with 4’,6-diamidino-2-phenylindole (DAPI) (blue). Scale bar, 10 μm. **(D)** Cytoplasm (CF) and nuclear (NF) fractions isolated from BmN cells expressing FLAG-TCTP-WT or FLAG-TCTP-K164R were probed with an anti-FLAG antibody. An anti-Tubulin antibody was used to label CF.

It has been shown that the human HsTCTP can be SUMOylated at a lysine 164 site (32). In order to identify whether the SUMOylation of TCTP is conserved in other species, we here investigated the potential SUMOylation target in silkworm. A comparison of the TCTP sequences from human and silkworm showed very high similarity in full-length and possessed a conserved lysine at 164 site (**Figure 1A**). To verify the SUMOylation, we constructed wild-type (WT) and mutant (K164R) overexpression plasmids of FLAG-TCTP-WT and FLAG-TCTP-K164R. These plasmids were respectively co-transfected with SUMOylation molecular HA-SMT3 into the silkworm BmN cells. The results of Co-IP analysis showed that TCTP-WT had an obvious band of SUMOylation compared with TCTP-K164R (**Figure 1B**). These findings suggested that the lysine residue at 164 was also the SUMOylation accept site of silkworm *BmTCTP*, which was consistent with the human HsTCTP (32).

We next analyzed if the SUMOylation mutation of *BmTCTP* would affect its subcellular localization. Red-tagged TCTP-WT and TCTP-K164R were transiently transfected into the BmN cells, respectively. As shown in **Figure 1C**, WT *BmTCTP* was primarily localized in the cytoplasm, indicating that silkworm *BmTCTP* was a cytoplasmic protein, which further confirmed the previous report (50). Also, K164R mutant *BmTCTP* showed similar cytoplasm localization as WT, implying that the SUMOylation mutation of *BmTCTP* did not affect its localization under the normal state of cultured cells. In agreement with the fluorescence signals, isolation of the cytoplasm and nuclear fractions of BmN cells showed that either TCTP-WT or TCTP-K164R was present in the cytoplasm by immunoblotting analysis (**Figure 1D**). Taken together, these data demonstrated that *BmTCTP* is SUMOylated at a conserved lysine site in silkworm and that the SUMOylation of *BmTCTP* did not influence its cytoplasm localization.

### *BmTCTP* Knockdown Promoted Viral Replication

**Figure 2:**
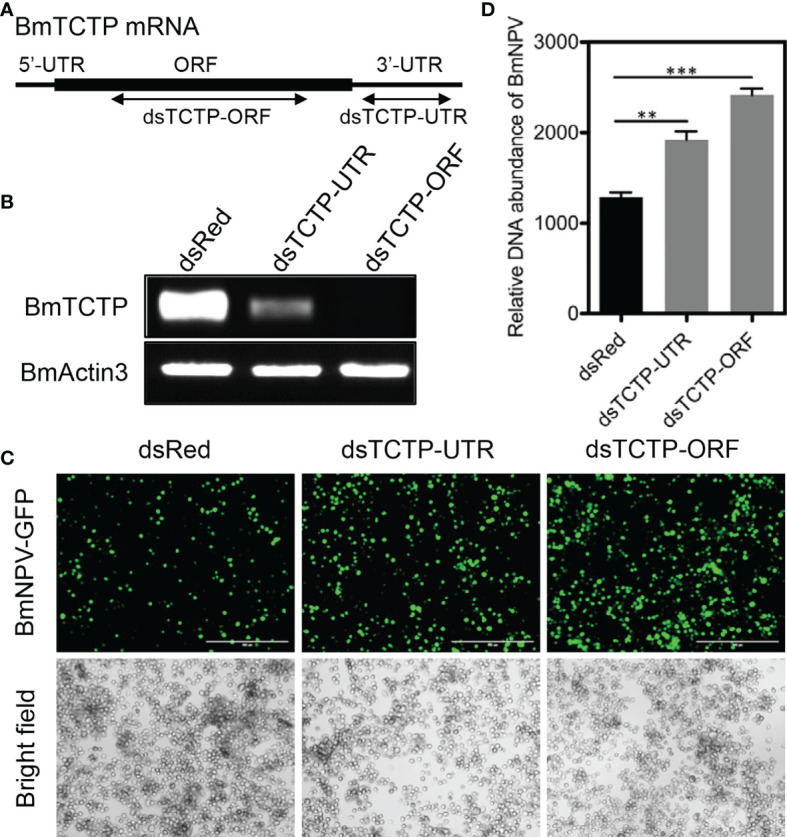
*Bombyx mori* translationally controlled tumor protein (*BmTCTP*) knockdown promoted viral replication. **(A)** Schematic representation of the silkworm *BmTCTP* gene structure and dsRNA target regions. **(B)** Semiquantitative RT-PCR was used to evaluate RNAi efficiency of dsRNA targeting TCTP-ORF and TCTP-UTR in cultured silkworm BmN cells. The expression of *BmActin3* gene was used as an internal control. **(C)** Fluorescence microscopy was used to observe the viral amounts when BmN cells were infected with *Bombyx mori nucleopolyhedrovirus* (BmNPV)-GFP for 72 h in the presence or absence of *BmTCTP*. Scale bar, 400 μm. **(D)** The viral DNA contents were detected by quantitative RT-PCR analysis of the *BmNPV GP41* gene. The expression of *BmGAPDH* gene was used as an internal control. The data presented are the means ± SD (n = 3). For the significant analysis: ^**^P < 0.01 and ^***^P < 0.001.

TCTP has been reported to be involved in the resistance against bacteria or viruses, and silencing of *BmTCTP* expression resulted in a decrease in survival rate of silkworm individuals after bacterial infection (38). To gain further insight into the function of *BmTCTP* in immune response, we here investigated its immune function in response to BmNPV infection by RNAi-mediated knockdown of *BmTCTP*. We firstly synthesized specific dsRNAs against ORF and UTR of *BmTCTP* (referred to as dsTCTP-ORF and dsTCTP-UTR) (**Figure 2A**). G5-PAMAM system was used to deliver dsRNAs of *BmTCTP* into the BmN cells to silence *BmTCTP* expression (45). RT-PCR result showed that both dsTCTP-ORF and dsTCTP-UTR were able to decrease the mRNA transcription of *BmTCTP* compared with the dsRed control treatment (**Figure 2B**), and also dsTCTP-ORF could specifically decrease the expression of FLAG-TCTP by immunoblotting (**Figure S1**), which together revealed that dsRNAs of *BmTCTP* could efficiently silence its own expression.

Then, BmNPV infection in the BmN cells was evaluated after *BmTCTP* knockdown. BmN cells were infected with BmNPV-GFP when the expression of *BmTCTP* was silenced. As shown in **Figure 2C**, fluorescence observation exhibited that the viral fluorescence was obviously stronger in cells of *BmTCTP* knockdown than that of the control. To further quantify the viral amounts, the viral DNA contents were detected by qRT-PCR analysis of the BmNPV *GP41* gene and compared with the control. Low levels of DNA replication were observed in control dsRNA treatment, and knockdown of *BmTCTP* led to a significant increase of viral amounts (**Figure 2D**). These data suggested that *BmTCTP* knockdown promoted the replication of BmNPV, indicating that the silkworm *BmTCTP* participated in the host immune response against virus infection.

### Deficiency of *BmTCTP* SUMOylation Inhibited Its Antiviral Immunity

**Figure 3:**
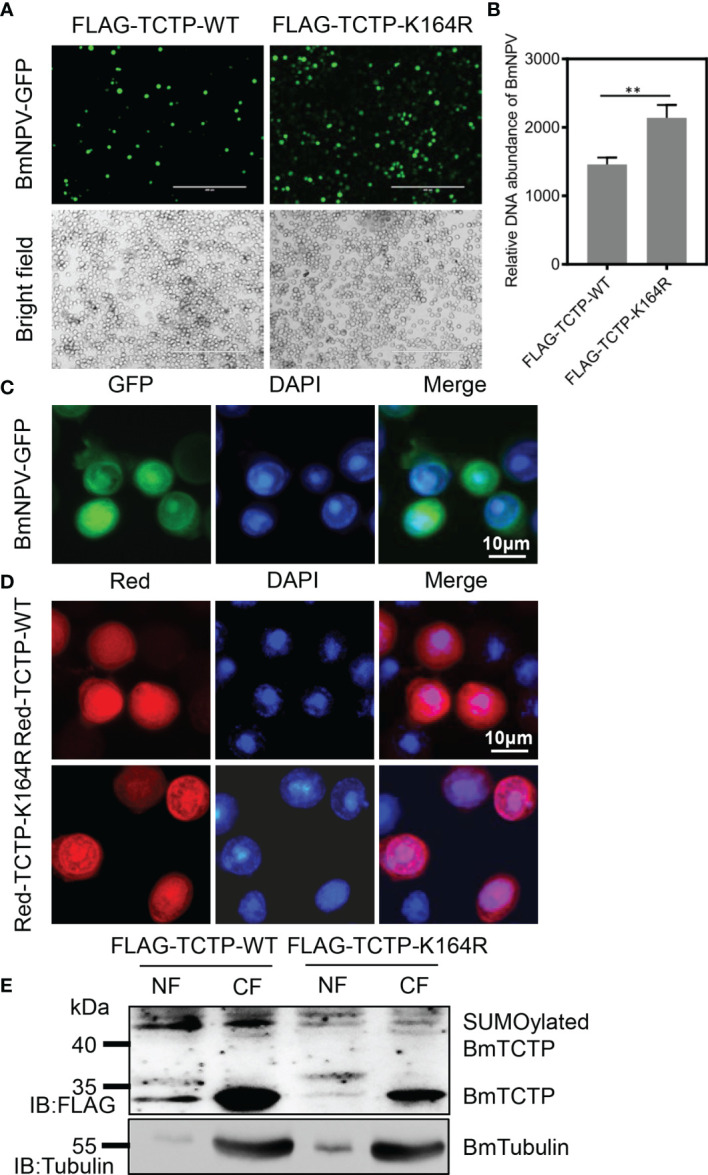
Deficiency of *Bombyx mori* translationally controlled tumor protein (*BmTCTP*) SUMOylation affected its antiviral immunity. **(A)** Plasmids as indicated were transfected into the silkworm BmN cells, and then cells were infected with *Bombyx mori nucleopolyhedrovirus* (BmNPV)-GFP for 72 h. Fluorescence microscopy was used to observe the viral amounts. Scale bar, 400 μm. **(B)** The viral DNA contents were detected by qRT-PCR analysis of the *BmNPV GP41* gene. The expression of *BmGAPDH* gene was used as an internal control. The data presented are the means ± SD (n = 3). For the significant analysis: ^**^P < 0.01. **(C)** BmN cells were infected with BmNPV-GFP for 72 h. BmNPV-positive cells were then determined by fluorescence (green), and the nuclear DNA was counterstained with 4’,6-diamidino-2-phenylindole (DAPI) (blue). Scale bar, 10 μm. **(D)** Red-TCTP-WT or Red-TCTP-K164R plasmid was transfected into the silkworm BmN cells, and then cells were infected with BmNPV-GFP for 72 h. Subcellular localization of Red-TCTP-WT or Red-TCTP-K164R in BmNPV-positive cells were determined by fluorescence (red), and the nuclear DNA was counterstained with DAPI (blue). Scale bar, 10 μm. **(E)** Cytoplasm (CF) and nuclear (NF) fractions isolated from BmNPV-GFP-infected cells expressing FLAG-TCTP-WT or FLAG-TCTP-K164R were probed with an anti-FLAG antibody. An anti-Tubulin antibody was used to label CF.

To investigate whether SUMOylation of *BmTCTP* was involved in the immune function to virus infection, we firstly transfected two plasmids expressing FLAG-TCTP-WT and FLAG-TCTP-K164R, respectively, into the BmN cells. After the challenge by BmNPV-GFP, viral fluorescence clearly showed that the expression of mutated TCTP-K164R induced higher BmNPV levels than that of TCTP-WT expression (**Figure 3A**). Consistent with this observation, qRT-PCR for *GP41* gene in TCTP-K164R also exhibited high levels of the viral DNA contents compared with TCTP-WT (**Figure 3B**). These results may indicate the possibility that the mutant of *BmTCTP* SUMOylation site affected its own immune response.

As cells infected by BmNPV caused the nuclear chromosomes to aggregate (**Figure 3C**), we next analyzed the distribution of *BmTCTP* in BmN cells after BmNPV treatment. It was shown that the TCTP-WT could enter the nucleus wherein the fluorescence signals were very strong. When compared with the TCTP-WT, however, the localization of TCTP-K164R mutation in the nucleus was decreased (**Figure 3D**). To further verify this result, we isolated the cytoplasm and nuclear fractions to determine the localization of *BmTCTP*. As shown in **Figure 3E**, we can detect the TCTP-WT protein in both cytoplasm and nucleus; by contrast, only a weak signal of the TCTP-K164R can be detected in the nucleus, suggesting that TCTP-WT indeed entered the nucleus post BmNPV infection. Interestingly, we found very high levels of SUMOylation in the TCTP-WT, but not in the TCTP-K164R, which indicated that BmNPV infection may induce the modification of *BmTCTP* SUMOylation and the enrichment into the nucleus. Taken together, these data suggested that SUMOylation may contribute to antiviral immunity of *BmTCTP*.

### Stabilization of *BmTCTP* SUMOylation Enhanced Its Antiviral Immunity

**Figure 4:**
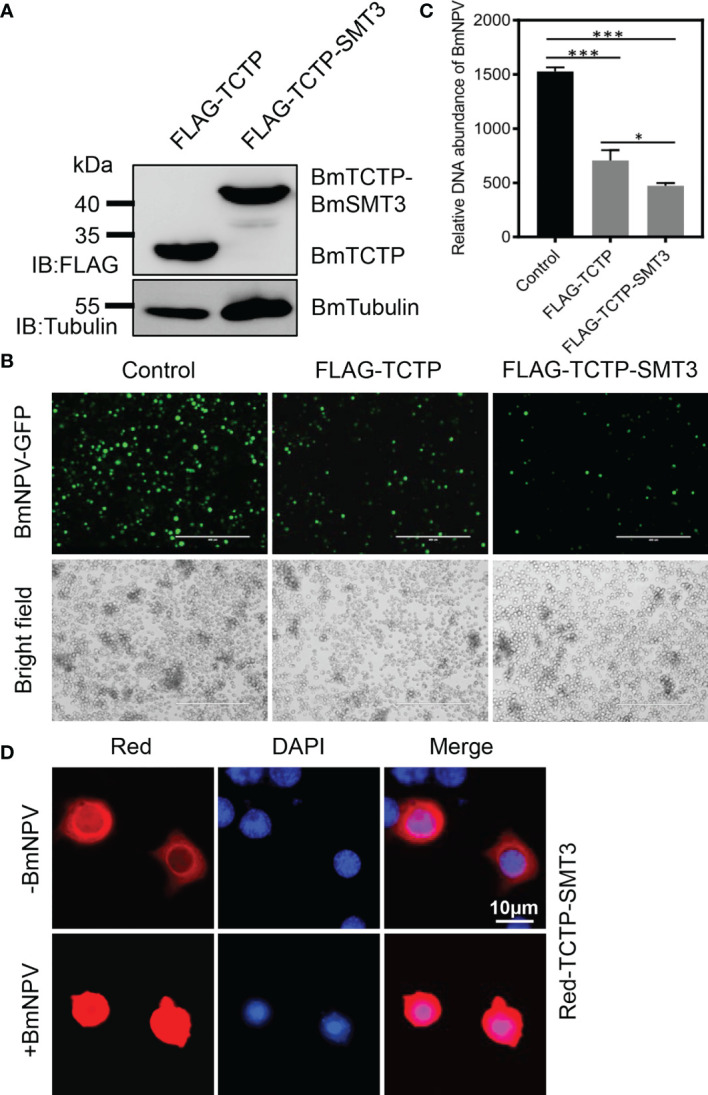
Stabilization of *Bombyx mori* translationally controlled tumor protein (*BmTCTP*) SUMOylation enhanced its antiviral immunity. **(A)** Plasmids expressing FLAG-TCTP and FLAG-TCTP-SMT3 were transfected into the silkworm BmN cells, and then cells were collected after 72 h. Proteins from FLAG-TCTP and FLAG-TCTP-SMT3 were detected by anti-FLAG and anti-Tubulin antibodies. **(B)** Plasmids as indicated were transfected into the silkworm BmN cells, and then cells were infected with *Bombyx mori nucleopolyhedrovirus* (BmNPV)-GFP for 72 h. Fluorescence microscopy was used to observe the viral amounts. Scale bar, 400 μm. **(C)** The viral DNA contents were detected by qRT-PCR analysis of the *BmNPV GP41* gene. The expression of *BmGAPDH* gene was used as an internal control. The data presented are the means ± SD (n = 3). For the significant analysis: ^*^P < 0.05 and ^***^P < 0.001. **(D)** Red-TCTP-SMT3 was transfected into the silkworm BmN cells, and then cells were infected with or without BmNPV-GFP for 72 h. Subcellular localization of Red-TCTP-SMT3 was determined by fluorescence (red), and the nuclear DNA was counterstained with 4’,6-diamidino-2-phenylindole (DAPI) (blue). Scale bar, 10 μm.

To further confirm that SUMOylation of *BmTCTP* was involved in the antiviral immunity, we next generated a fusion vector of *BmTCTP* and BmSMT3 proteins in order to maintain the state of *BmTCTP* SUMOylation. Expression of FLAG-TCTP-SMT3 was measured by immunoblotting, and the protein size of FLAG-TCTP-SMT3 was larger than that of FLAG-TCTP (**Figure 4A**), which also supported the SUMOylated *BmTCTP* in **Figure 1B**. We then transfected the FLAG-TCTP or FLAG-TCTP-SMT3 into the BmN cells infected with BmNPV-GFP, and post 72 h treatment, the viral fluorescence was observed. It was shown that the expression of *BmTCTP*-BmSMT3 obviously inhibited BmNPV levels compared with that of *BmTCTP* itself, although both treatments also reduced viral signals when compared with the control (**Figure 4B**). In agreement with this, qRT-PCR for *GP41* gene in *BmTCTP*-BmSMT3 also significantly decreased levels of the viral DNA contents compared with *BmTCTP* and control (**Figure 4C**). These results indicated that stabilization of *BmTCTP* SUMOylation indeed enhanced its antiviral function.

To detect the subcellular localization of *BmTCTP*-BmSMT3 upon the infection with or without BmNPV, Red-TCTP-SMT3 was transfected into cells to analyze its distribution. In the absence of BmNPV, Red-TCTP-SMT3 was mainly localized in the cytoplasm (**Figure 4D**), consistent with the WT *BmTCTP* localization (**Figure 1C**). By contrast, in the presence of BmNPV, Red-TCTP-SMT3 was able to largely accumulate in the nucleus (**Figure 4D**). Altogether, these data demonstrated that SUMOylation enabled *BmTCTP* to promote the immune response against the virus.

### SUMO Conjugating Enzyme BmUBC9 Was Required for the SUMOylation of *BmTCTP*

**Figure 5:**
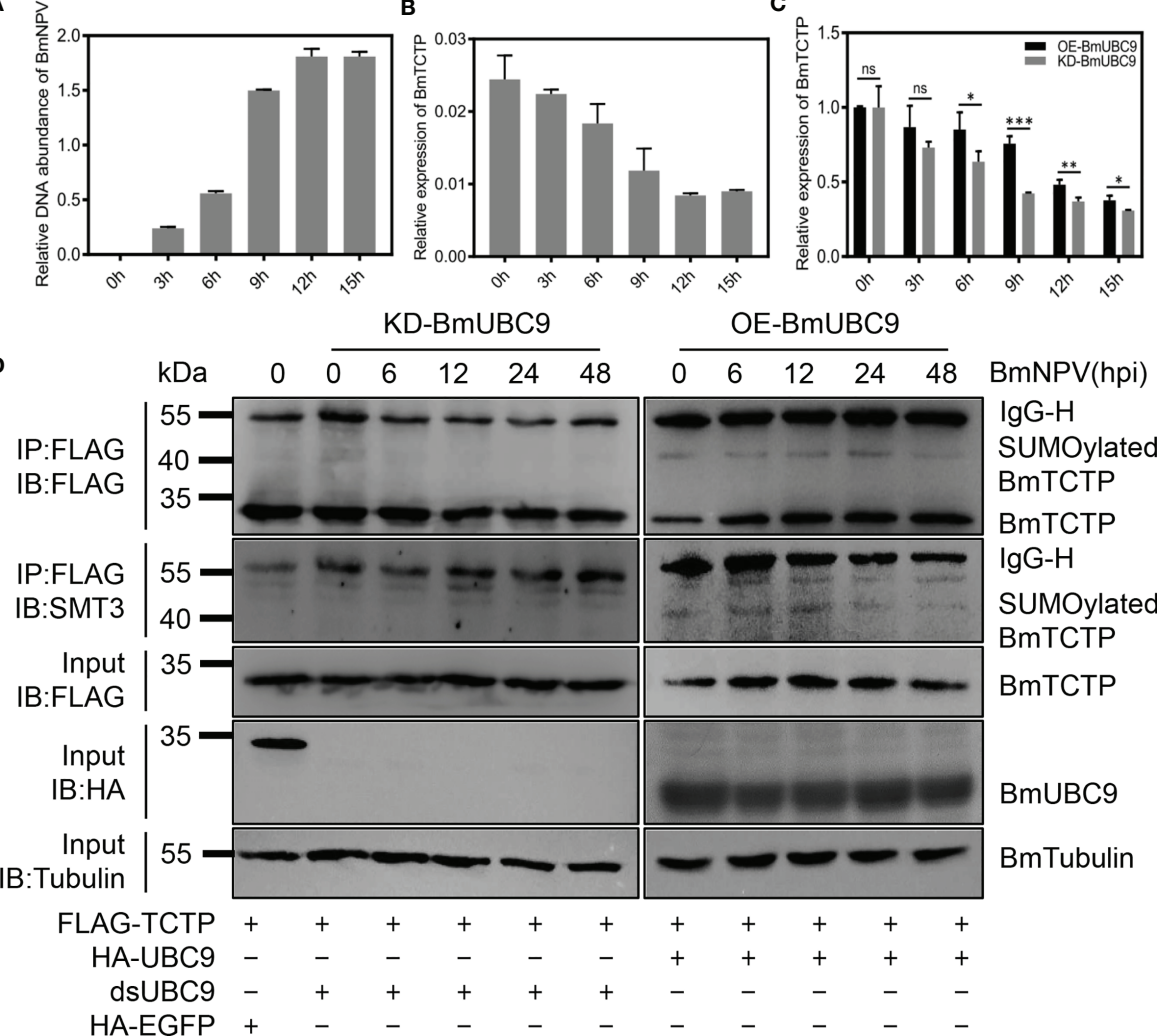
SUMO conjugating enzyme BmUBC9 was required for the SUMOylation of *Bombyx mori* translationally controlled tumor protein (*BmTCTP*). **(A)** BmN cells infected with *Bombyx mori nucleopolyhedrovirus* (BmNPV) were collected at the indicated time points and used for qRT-PCR analysis of the *BmNPV GP41* gene. The expression of *BmGAPDH* gene was used as an internal control. The data presented are the means ± SD (n = 3). **(B)** The expression of *BmTCTP* gene was analyzed by qRT-PCR following the BmNPV infection, and the BmeIF-4a was used as an internal control. The data presented are the means ± SD (n = 3). **(C)** The expression of *BmTCTP* gene was analyzed by qRT-PCR in BmUBC9 knockdown or overexpression cells following the BmNPV infection, and the BmeIF-4a was used as an internal control. The data presented are the means ± SD (n = 3). For the significant analysis: ^*^P < 0.05, ^**^P < 0.01, and ^***^P < 0.001, and ns represents no significance. **(D)** Plasmids as indicated were co-transfected into the BmN cells infected with BmNPV. Lysates from BmN cells were used for immunoprecipitation with anti-FLAG antibody, followed by immunoblotting with the indicated antibodies. SUMOylated *BmTCTP* was further determined by an anti-SMT3 antibody. IgG-L and IgG-H were the light and heavy chains of anti-FLAG antibody.

To address how SUMOylation pathway was involved in the *BmTCTP* SUMOylation and immune response, we investigated the potential role of BmUBC9, a SUMO conjugating enzyme responsible for all SUMO conjugations in eukaryotic cells (42, 51, 52), in the *BmTCTP* SUMOylation. We firstly monitored the DNA amounts of BmNPV infection in cells at the indicated time points. It was shown that following the virus infection, BmNPV replications were increased rapidly (**Figure 5A**). By contrast, the expression of *BmTCTP* was downregulated after BmNPV infection (**Figure 5B**). These results suggested that the virus might inhibit the immune function of *BmTCTP* by downregulating *BmTCTP* expression levels in the host cells and thus prompted its own replications.

It was interesting that when we knocked down or overexpressed the BmUBC9 in the BmN cells infected with BmNPV, the decrease of *BmTCTP* expression was delayed in BmUBC9 overexpression cells compared with that in BmUBC9 knockdown cells (**Figure 5C**), implying the possibility that BmUBC9 could be involved in the immune response of *BmTCTP*. To test this, we carried out the immunoprecipitation analysis to analyze the SUMOylation levels of *BmTCTP* in BmUBC9 knockdown or overexpression cells upon BmNPV infection. As shown in **Figure 5D**, deletion of BmUBC9 clearly disrupted the SUMOylation of *BmTCTP*, whereas overexpression of BmUBC9 allowed cells to maintain high levels of *BmTCTP* SUMOylation even in the cells infected with a long period of BmNPV. Hence, the present data indicated that BmUBC9 would be essential for *BmTCTP* SUMOylation as well as its stabilization.

### *BmTCTP* Interactome Identified the Interaction With an Immune Molecule Interleukin Enhancer Binding Factor

**Figure 6:**
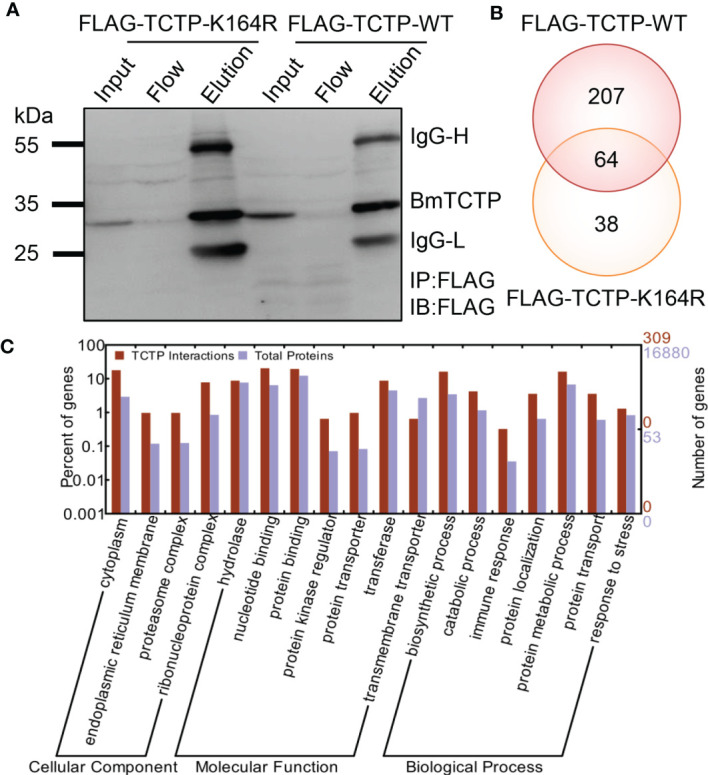
*Bombyx mori* translationally controlled tumor protein (*BmTCTP*) interactome identified the interaction with an immune molecule *Bombyx mori* interleukin enhancer binding factor (*BmILF*). **(A)** The efficiency of anti-FLAG antibody-mediated *BmTCTP* immunoprecipitation. Anti-FLAG antibody was used for both IP and immunoblotting (IB). The input, flow, and elution proteins were resolved on 10% SDS-PAGE gels and probed with anti-FLAG antibody. IgG-L and IgG-H were the light and heavy chains of anti-FLAG antibody. **(B)** Venn diagram assay of the FLAG-TCTP-WT and FLAG-TCTP-K164R interacting proteins identified by liquid chromatography-tandem mass spectrometry (LC-MS/MS). The LC-MS/MS data for each expression plasmid were shown in **Table S2**. **(C)** Functional categories of the interacting proteins were analyzed by WEGO. Proteins were classified into cellular component, molecular function, and biological process according to their Gene Ontology (GO) signatures, and the total silkworm genes were shown as a reference.

To better understand how *BmTCTP* participates in the immune process, we next aimed to identify proteins interacting with *BmTCTP* in BmN cells. We established two cell lines stably expressing FLAG-TCTP-WT and FLAG-TCTP-K164R, respectively. Whole-cell lysates were prepared from both cell lines, and FLAG antibody against FLAG peptide was used for immunoprecipitation in the above cell lysates. The resulting bound or flow proteins were resolved on 10% SDS-polyacrylamide gel electropheresis (SDS-PAGE) gels and probed with anti-FLAG antibody (**Figure 6A**). The eluate after the elution was further applied to SDS-PAGE, and the peptides were generated *via* in-gel trypsin digestion and subjected to LC-MS/MS analysis. As a result, we obtained 271 interaction proteins in FLAG-TCTP-WT and 102 proteins in FLAG-TCTP-K164R, and there were 64 common proteins (**Figure 6B**). These differential interactomes between WT and K164R mutant indicated that the SUMOylation of *BmTCTP* may contribute to its own interaction with other proteins.

To classify the identified *BmTCTP* interacting proteins according to biological processes, molecular functions, or cellular component, we performed a Gene Ontology (GO) analysis to annotate protein functions. The resulting GO terms were used to categorize the proteins using WEGO program. It was shown that the majority of proteins will function in protein binding, as a protein kinase regulator, and as a protein transporter and participate in immune response and protein localization in silkworm (**Figure 6C**), suggesting that the silkworm *BmTCTP* would also act as a multifunctional protein through binding directly or indirectly to a number of proteins. Interestingly, among the interaction proteins, we found that only one immune molecule called ILF was just presented in TCTP-WT interactome but not in TCTP-K164R (**Table S2**), which implied that the *BmILF* may be involved in the immune function of *BmTCTP*.

### *BmTCTP* Regulated *BmILF* Expression and Inhibited Viral Replication

**Figure 7:**
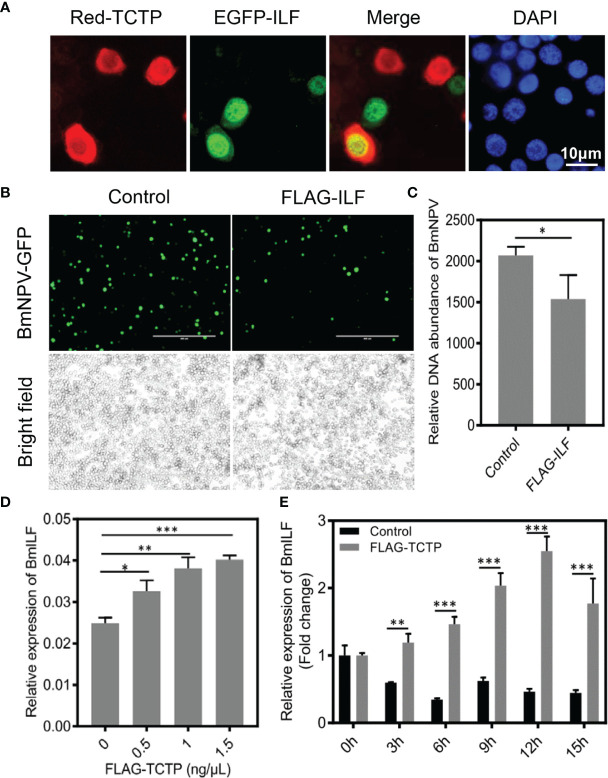
*Bombyx mori* translationally controlled tumor protein (*BmTCTP*) regulated *Bombyx mori* interleukin enhancer binding factor (*BmILF*) expression and inhibited viral replication. **(A)** Plasmids encoding Red-TCTP and EGFP-ILF were co-transfected into the BmN cells. After 3 days of transfection, cells were fixed and stained with 4’,6-diamidino-2-phenylindole (DAPI) (blue). Scale bar, 10 μm. **(B)** Plasmids as indicated were transfected into the BmN cells, and then cells were infected with *Bombyx mori nucleopolyhedrovirus* (BmNPV)-GFP for 72 h. Fluorescence microscopy was used to observe the viral amounts. Scale bar, 400 μm. **(C)** The viral DNA contents were detected by qRT-PCR analysis of the *BmNPV GP41* gene. The expression of *BmGAPDH* gene was used as an internal control. The data presented are the means ± SD (n = 3). For the significant analysis: ^*^P < 0.05. **(D)** The expression of *BmILF* gene was analyzed by qRT-PCR in *BmTCTP* overexpression cells at different concentrations, and the BmeIF-4a was used as an internal control. The data presented are the means ± SD (n = 3). For the significant analysis: ^*^P < 0.05, ^**^P < 0.01, and ^***^P < 0.001. **(E)** The fold changes of *BmILF* expression was analyzed by qRT-PCR in *BmTCTP* overexpression cells following the BmNPV infection at the indicated time points, and the BmeIF-4a was used as an internal control. The data presented are the means ± SD (n = 3). For the significant analysis: ^**^P < 0.01 and ^***^P < 0.001.

Indeed, previous studies have demonstrated that human interleukin enhancer binding factor 2 (ILF2) is considered as an important host cellular protein, which exerts a negatively regulatory effect on the replication of various viruses, such as human immunodeficiency virus type 1 (HIV-1), porcine reproductive and respiratory syndrome virus (PRRSV), and Japanese encephalitis virus (JEV) (53–57). To further characterize the correlation between *BmTCTP* and *BmILF* and whether the silkworm *BmILF* was able to regulate BmNPV replication, we investigated their functions in the response to BmNPV infection. It has been demonstrated that TCTP-WT was able to interact with *BmILF* by IP experiment. We further analyzed their interaction by colocalization. As shown in **Figure 7A**, *BmILF* was primarily localized in the nucleus, and only a small part of *BmILF* in the cytoplasm could be colocalized with *BmTCTP*.

To study the response of *BmILF* to the virus infection, we overexpressed *BmILF* in the BmN cells and infected BmNPV-GFP for 72 h. The viral fluorescence clearly showed that the expression of FLAG-ILF inhibited BmNPV compared with the control (**Figure 7B**). FLAG-ILF expression also significantly decreased levels of the viral DNA contents compared with the control by qRT-PCR for *GP41* gene (**Figure 7C**). These results implied that the silkworm *BmILF* was able to negatively regulate BmNPV replication.

Interestingly, we found that the expression of *BmILF* could be induced by overexpressed *BmTCTP*; moreover, this increased expression of *BmILF* was dependent on the levels of *BmTCTP* (**Figure 7D**), which suggested a possibility that *BmTCTP* may regulate *BmILF* expression as well. Although the expression of *BmILF* was also decreased post-infection with BmNPV at the indicated time points, overexpression of *BmTCTP*, however, significantly upregulated *BmILF* expression (**Figure 7E**). Therefore, our data demonstrated that *BmTCTP* will exert antiviral function *via* regulating *BmILF* expression and may in turn form an immune complex to inhibit the replication of virus in silkworm.

## Discussion

Increasing amount of evidence has proven that TCTP contributes to regulating immune function and plays important roles in diverse biological processes. In eukaryotes, TCTP has been considered as an antibacterial or antiviral factor. For example, RNAi of *Drosophila melanogaster* TCTP increased the mortality after oral infection with *Serratia marcescens* (37). The silence of TCTP in *Litopenaeus vannamei* led to the increase of viral replication of white spot syndrome virus (WSSV), and injection of purified TCTP into WSSV-infected *Penaeus monodon* increased its survival rate (58). RNAi of the silkworm TCTP inhibited the antimicrobial capacity and the intestinal innate immunity of the silkworm after oral infection of bacteria (38). Although the immune response of TCTP is widely accepted, its regulatory mechanisms are still largely unknown.

In the present study, we established that the silkworm *BmTCTP* could be modified by SUMOylation molecular BmSMT3 at the lysine 164, and the SUMOylated *BmTCTP* would contribute to its immune response and promote antiviral activity. Our data showed that *BmTCTP* RNAi led to increased susceptibility of the silkworm cells to virus infection, and by contrast, overexpression of *BmTCTP* significantly decreased the virus replication, implying a strong antiviral function of *BmTCTP* in silkworm. Unexpectedly, this antiviral function of *BmTCTP* had been attenuated by mutation of *BmTCTP* SUMOylation site, suggesting that the SUMOylation of *BmTCTP* would be required for the survival of the silkworm cells upon the virus challenge. Consistent with this, it was interestingly shown that the stabilization of *BmTCTP* SUMOylation by overexpressing *BmTCTP*-BmSMT3 fusion protein further suppressed the replication of the virus. Hence, the enhanced immune response of *BmTCTP* SUMOylation would be fascinating to evaluate the antiviral function in silkworm individuals. Recent work also showed that *Drosophila* Relish can be SUMOylated, and this SUMOylation prevents cleavage and activation of Relish, thus negatively regulating the immune response (59). All these data suggested that SUMOylation status in organisms would exactly contribute to differential immune response dependent on the different substrate proteins.

Furthermore, our results showed that BmUBC9 can act as a SUMO conjugating enzyme for *BmTCTP* SUMOylation. After the virus infection, knockdown or overexpression of UBC9 displayed differential SUMOylation status of *BmTCTP*. It was also shown that the decreased expression of *BmTCTP* following the virus challenge was partially rescued by overexpression of BmUBC9 rather than its knockdown, which implied that BmUBC9 may regulate *BmTCTP* expression as well. Future research is needed to improve our understanding of the molecular mechanisms underlying the BmUBC9-mediated regulation of *BmTCTP* promoter activity. However, it was demonstrated that the BmUBC9-mediated *BmTCTP* SUMOylation would be essential for antiviral function.

In order to decipher the immune function of *BmTCTP*, we also performed a systematic proteomics study by LC-MS/MS analysis in combination with IP to investigate its interaction proteins. As a result, a total of 309 potential *BmTCTP* binding proteins have been identified. The protein functional classification system showed that these proteins participate in various functional processes including protein binding, protein kinase regulation, protein transport, immune response, and protein localization, which is consistent with the reports of previous studies (17, 18). It was intriguing that the mutant of TCTP-K164R clearly affected the interaction of *BmTCTP* with a majority of target proteins, suggesting that the SUMOylation of *BmTCTP* would be also important for the interactions. Moreover, SUMO-activating enzyme was also specifically pulled down by TCTP-WT rather than by TCTP-K164R, further confirming the presence of SUMOylation in *BmTCTP* protein, which together provides a novel insight into deciphering the *BmTCTP* functions by SUMOylation.

Among the *BmTCTP* interactions, it was shown that *BmILF*, the only one immune molecule, is able to interact with TCTP-WT but not with TCTP-K164R, which gives us an exciting clue to understand the immunity function of *BmTCTP*. The human ILF homologue has been demonstrated as an important host cellular protein that exerts a negatively regulatory effect on the replication of various viruses (53, 55, 57). Our result also showed that overexpression of the silkworm *BmILF* significantly inhibited virus replication. Importantly, *BmTCTP* could induce the expression of *BmILF*, especially in the response of BmNPV infection. Given the interaction between *BmTCTP* and *BmILF*, and the induced expression of *BmILF* by *BmTCTP*, it was interesting to speculate that *BmTCTP* may regulate the expression of *BmILF* and further form the complex with *BmILF* upon virus challenge so as to act antivirus roles. Nevertheless, the molecular mechanisms by which *BmTCTP* regulates *BmILF* expression and activity require further investigation.

Taken together, our study unravels a signaling pathway mediated by BmUBC9-mediated SUMOylation of *BmTCTP* that can inhibit the replication of BmNPV. Further downstream signaling in the antivirus activity of *BmTCTP* would be ascribed to by the regulation of *BmILF* interaction with *BmTCTP* and/or *BmILF* expression in response to virus infection. It has been shown that diseases caused by viruses, especially by BmNPV, are the greatest challenge to sericulture and usually cause major economic losses of cocoon production every year (39, 40). A better understanding of the silkworm immune response will be helpful for the disease control. Therefore, our present study has provided a novel strategy to defend against a virus *via* the antiviral capacity of *BmTCTP* SUMOylation in cultured silkworm cells. It will be of great interest to examine whether the transgenic expression of *BmTCTP* SUMOylation form in silkworm individuals plays a similar role in replication control of the virus, which would be useful for generating antiviral silkworm lines in the future.

## Data Availability Statement

The original contributions presented in the study are included in the article/**Supplementary Material**. Further inquiries can be directed to the corresponding author.

## Author Contributions

ZL, QX, and PZ contributed to conception and design of the study. CL and ZL wrote the article. CL, ZL, WZ, HG, WL, and GS performed the research and analyzed the data. All authors contributed to the article and approved the submitted version.

## Funding

This work was supported by the National Natural Science Foundation of China (No. 32030103), Natural Science Foundation of Chongqing (Nos. cstc2020jcyj-cxttX0001 and cstc2021jsyj-yzysbAX0004), and Fundamental Research Funds for the Central Universities (No. XDJK2019B007).

## Conflict of Interest

The authors declare that the research was conducted in the absence of any commercial or financial relationships that could be construed as a potential conflict of interest.

## Publisher’s Note

All claims expressed in this article are solely those of the authors and do not necessarily represent those of their affiliated organizations, or those of the publisher, the editors and the reviewers. Any product that may be evaluated in this article, or claim that may be made by its manufacturer, is not guaranteed or endorsed by the publisher.

## Supplementary Material

Supplementary Figure 1 RNAi efficiency of dsRNA for *BmTCTP* by immunoblotting analysis. Immunoblotting was used to evaluate RNAi efficiency of dsRNA targeting TCTP-ORF and TCTP-UTR in FLAG-TCTP expressing BmN cells. Proteins from different treatments were isolated and detected by anti-FLAG and anti-Tubulin antibodies.

Supplementary Table 2 Identification of *BmTCTP* interacting proteins by mass spectrometry in silkworm.
