# Supplementary figures and images for: SUMOylation of Translationally Regulated Tumor Protein Modulates Its Immune Function
Source: Front Immunol. 2022 Feb 7;13:807097. doi: 10.3389/fimmu.2022.807097 (PMC8858932; doi:10.3389/fimmu.2022.807097)

Figure S1

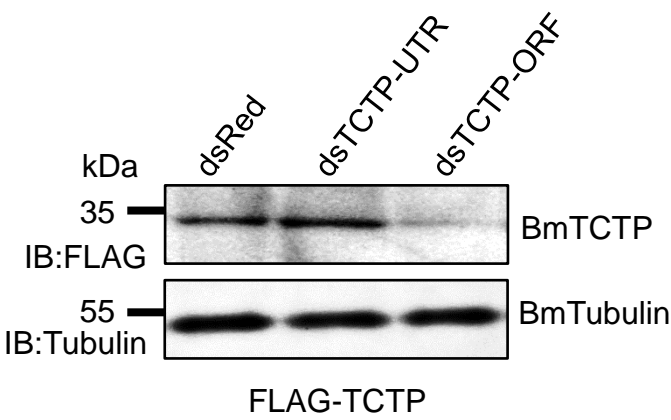

Supplement: Supplementary Figure 1 — RNAi efficiency of dsRNA for BmTCTP by immunoblotting analysis. Immunoblotting was used to evaluate RNAi efficiency of dsRNA targeting TCTP-ORF and TCTP-UTR in FLAG-TCTP expressing BmN cells. Proteins from different treatments were isolated and detected by anti-FLAG and anti-Tubulin antibodies. [file DataSheet_1.pdf]
