# Supplementary material for: SUMOylation of Translationally Regulated Tumor Protein Modulates Its Immune Function
Source: Front Immunol. 2022 Feb 7;13:807097. doi: 10.3389/fimmu.2022.807097 (PMC8858932; doi:10.3389/fimmu.2022.807097)

Related to Figure 1B

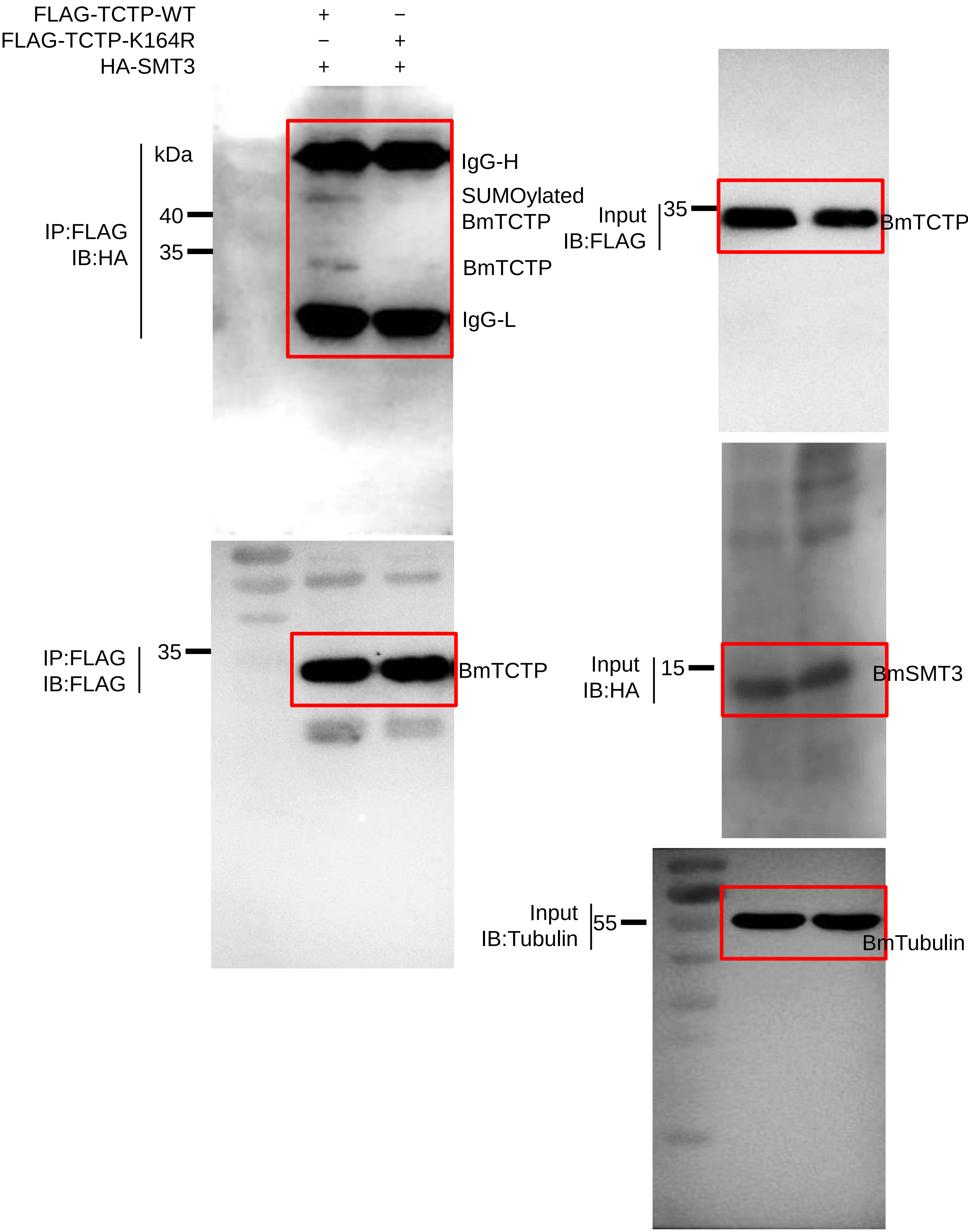

Related to Figure 1C

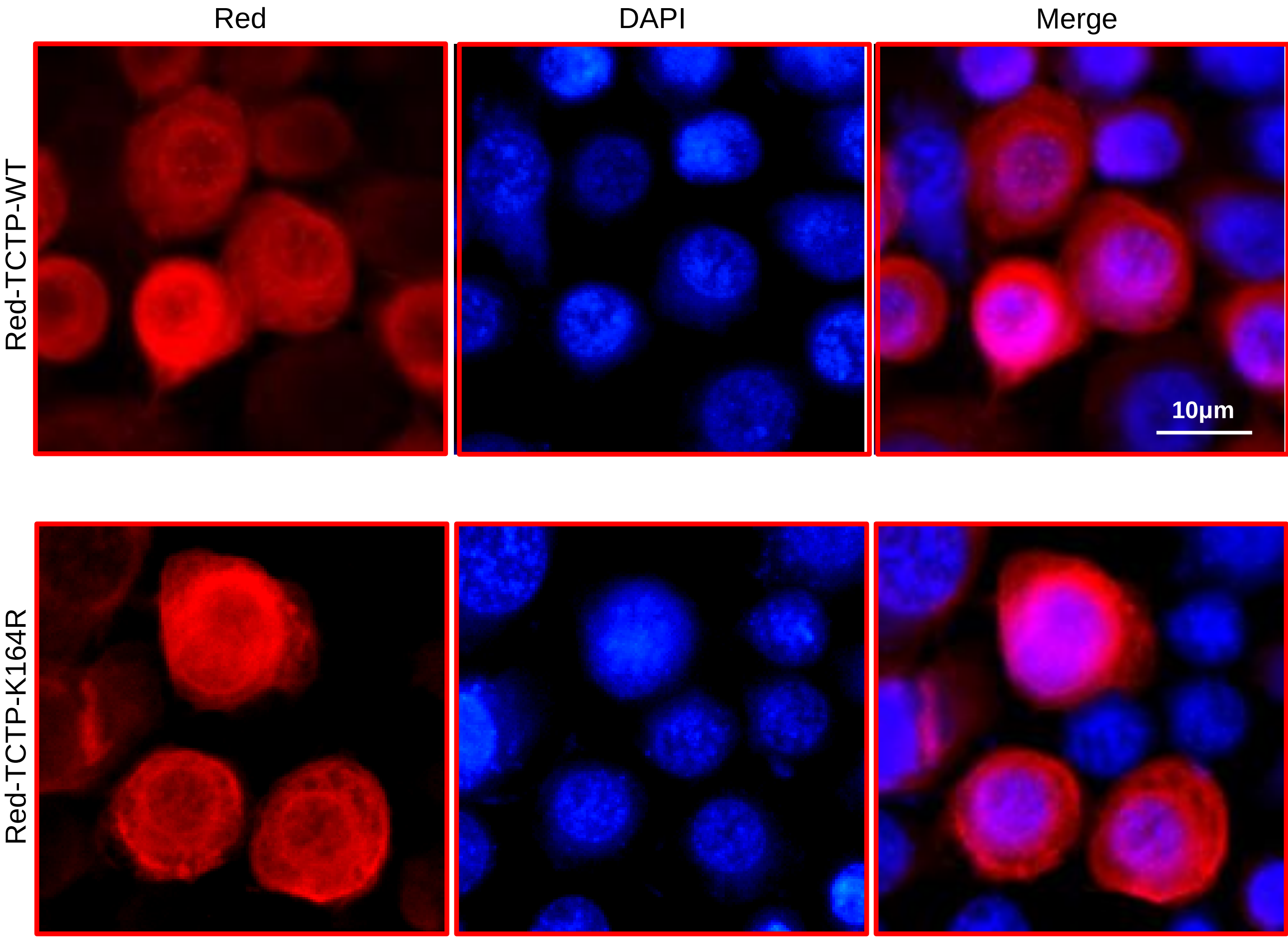

Related to Figure 1D

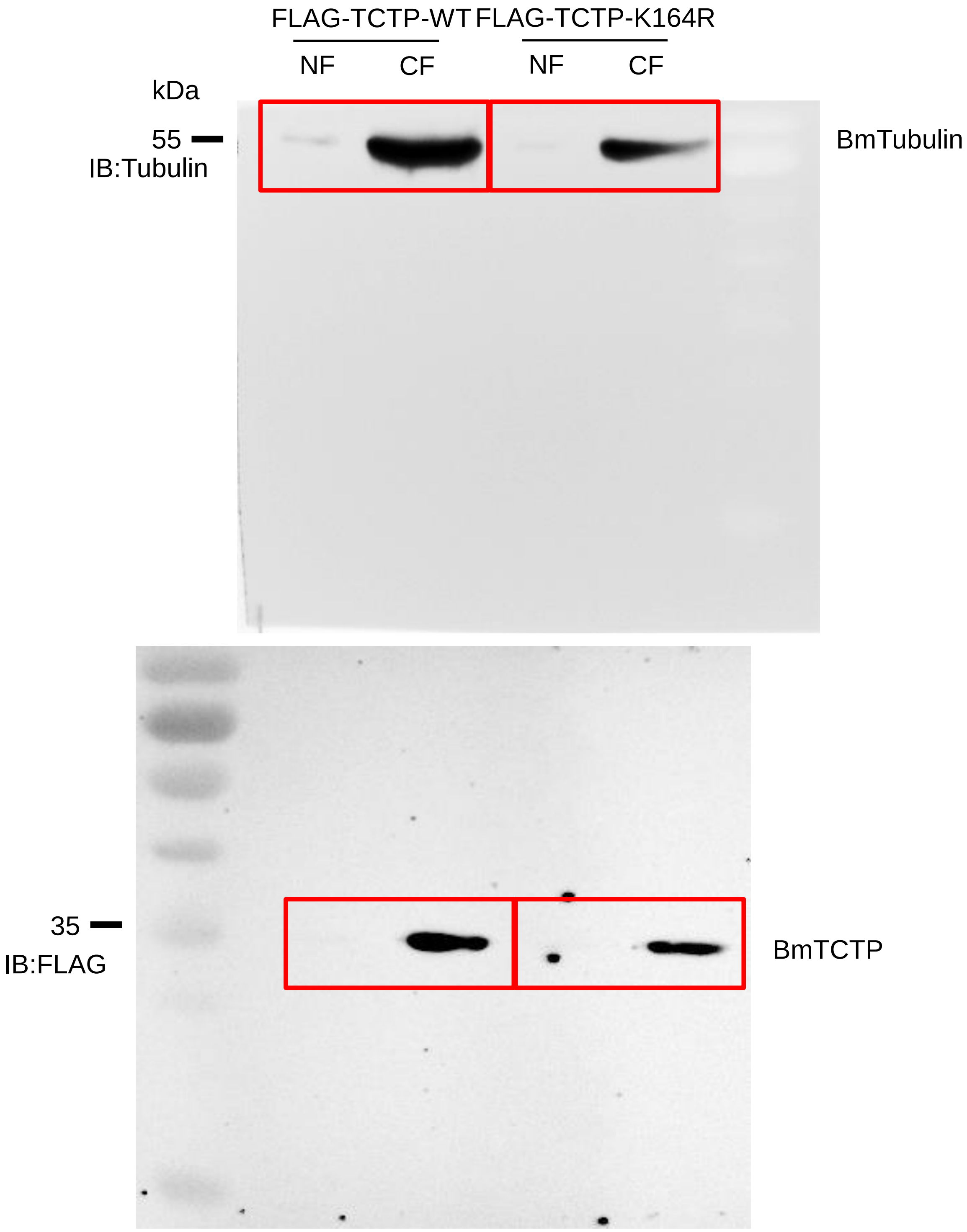

Related to Figure 2B

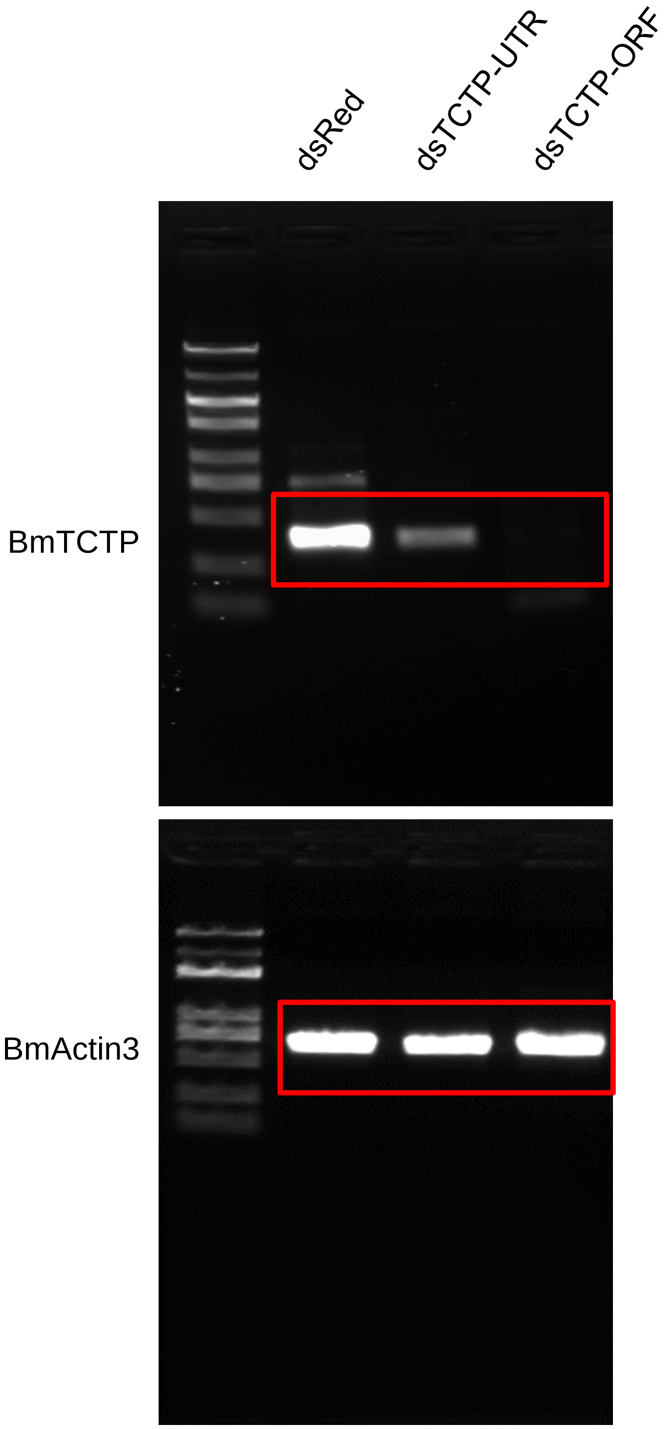

Related to Figure 2C

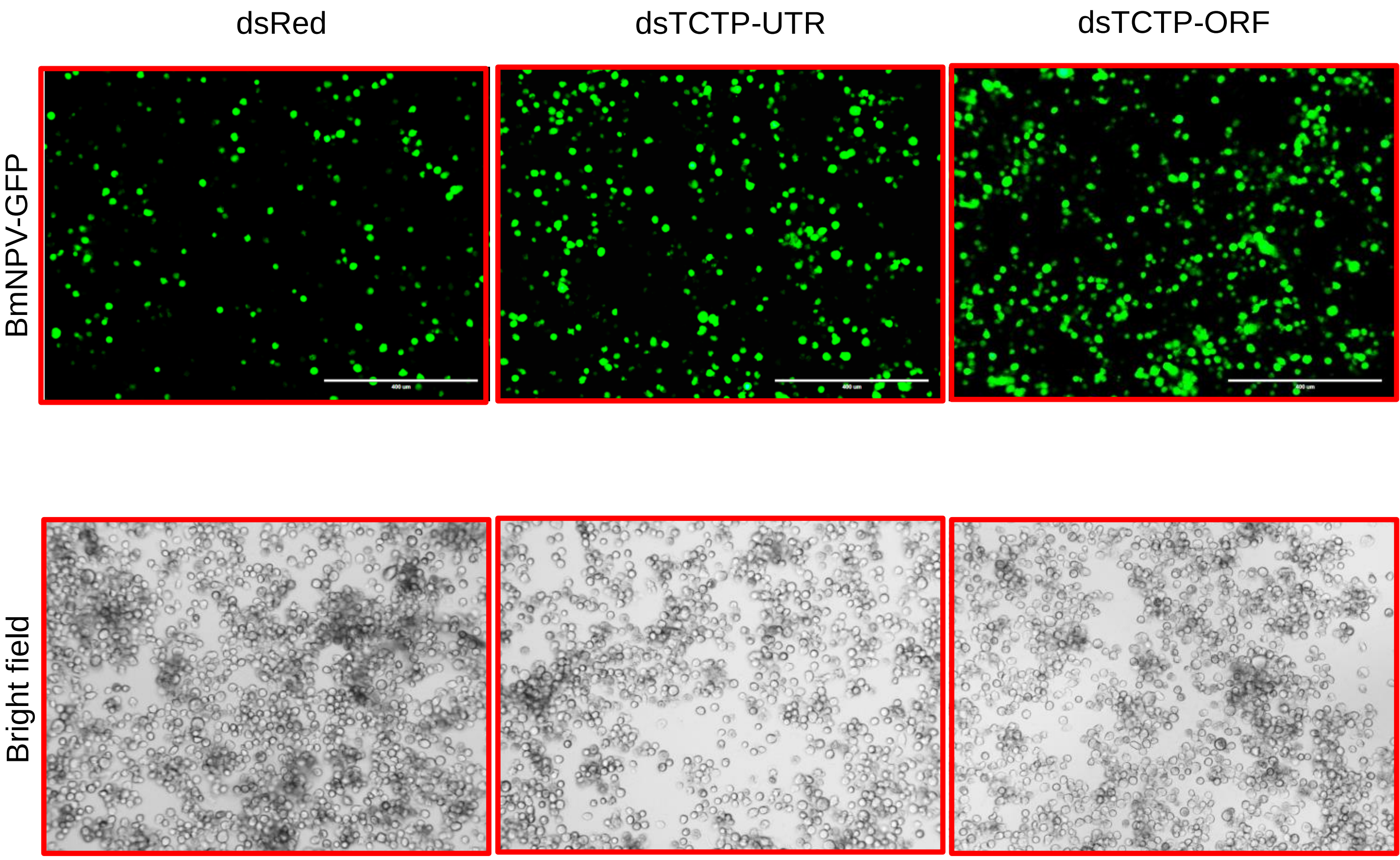

Related to Figure 3A

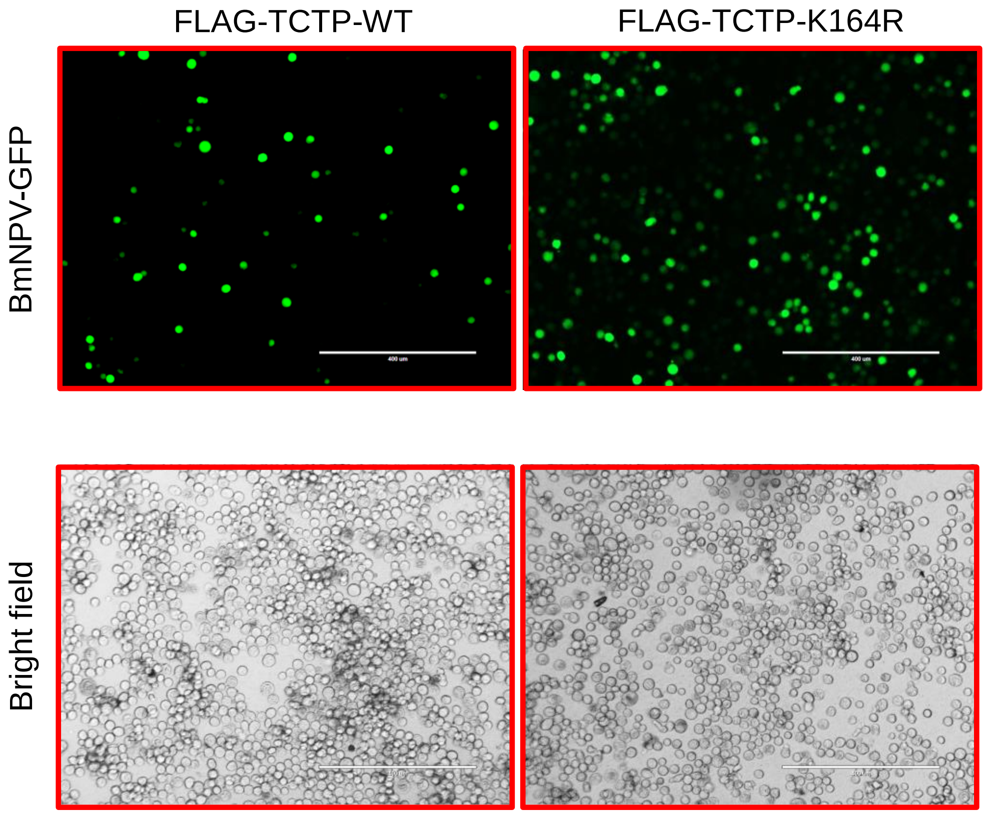

Related to Figure 3C

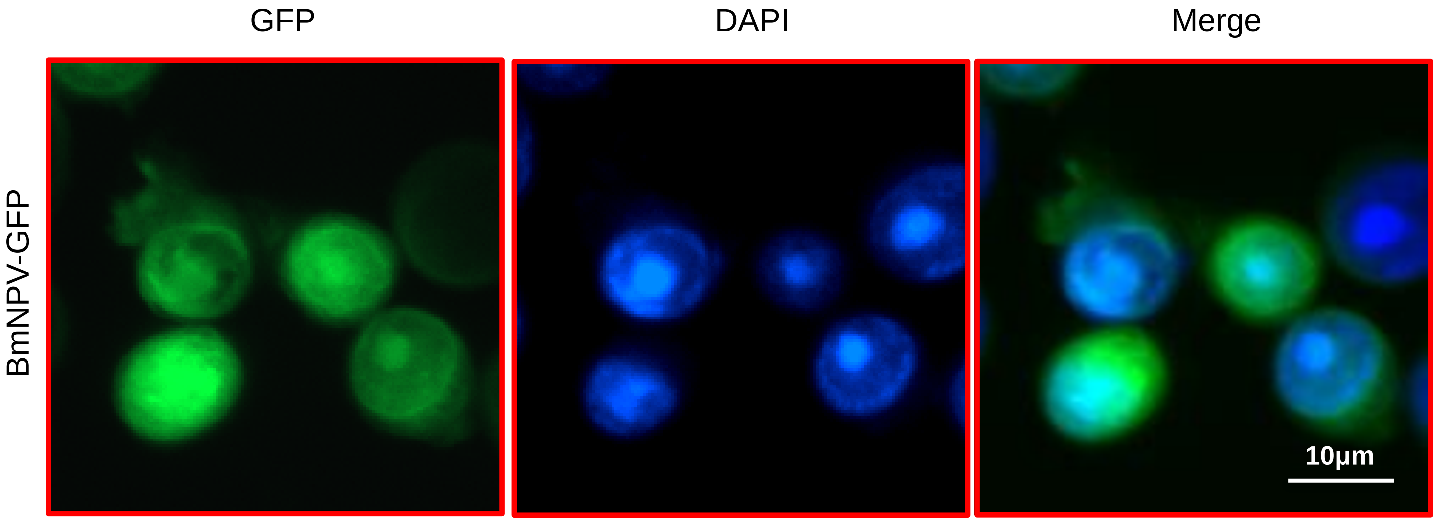

Related to Figure 3D

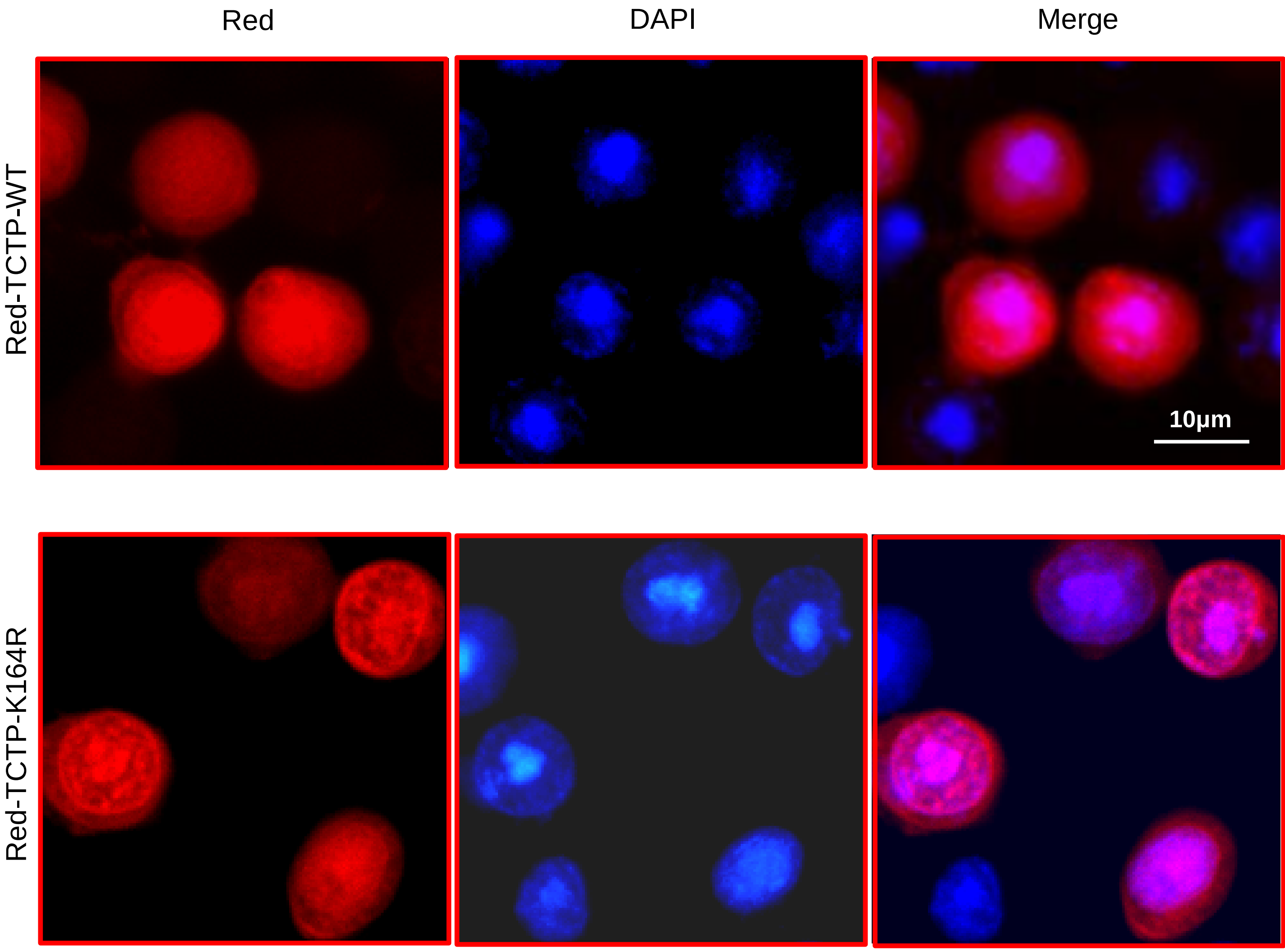

Related to Figure 3E

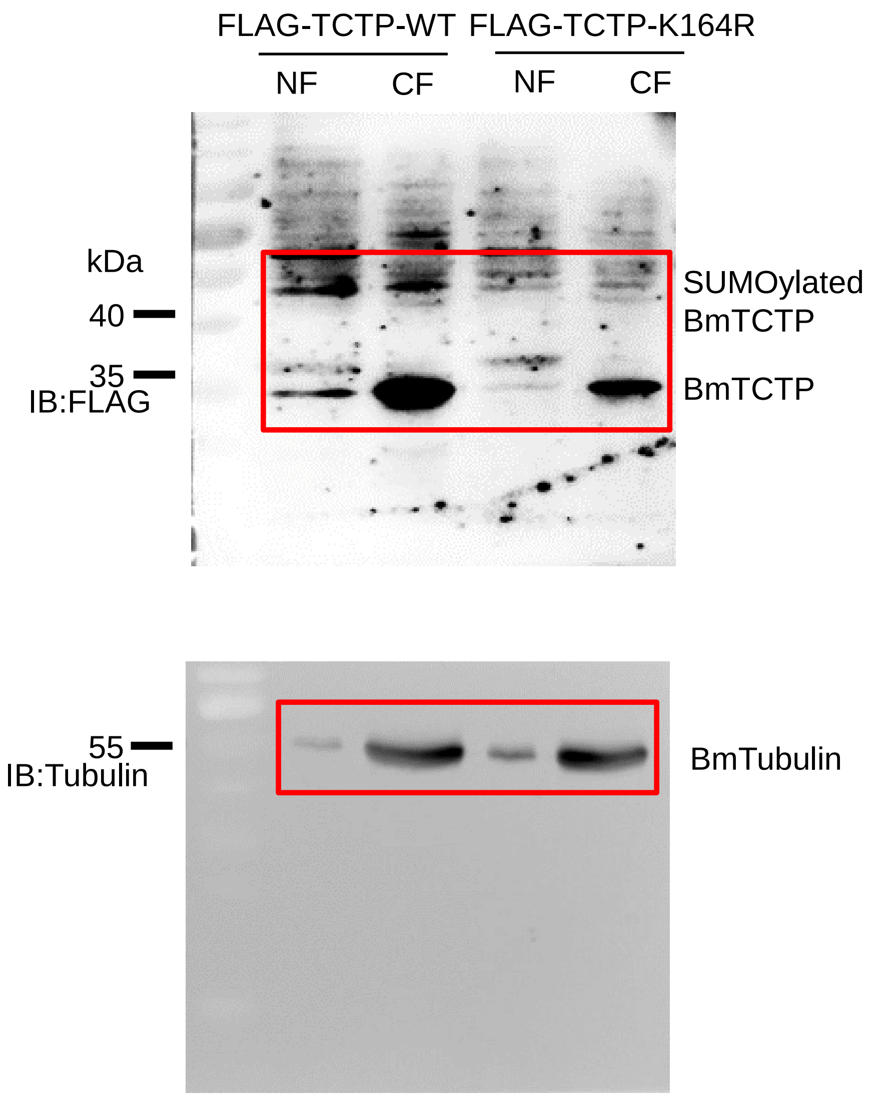

Related to Figure 4A

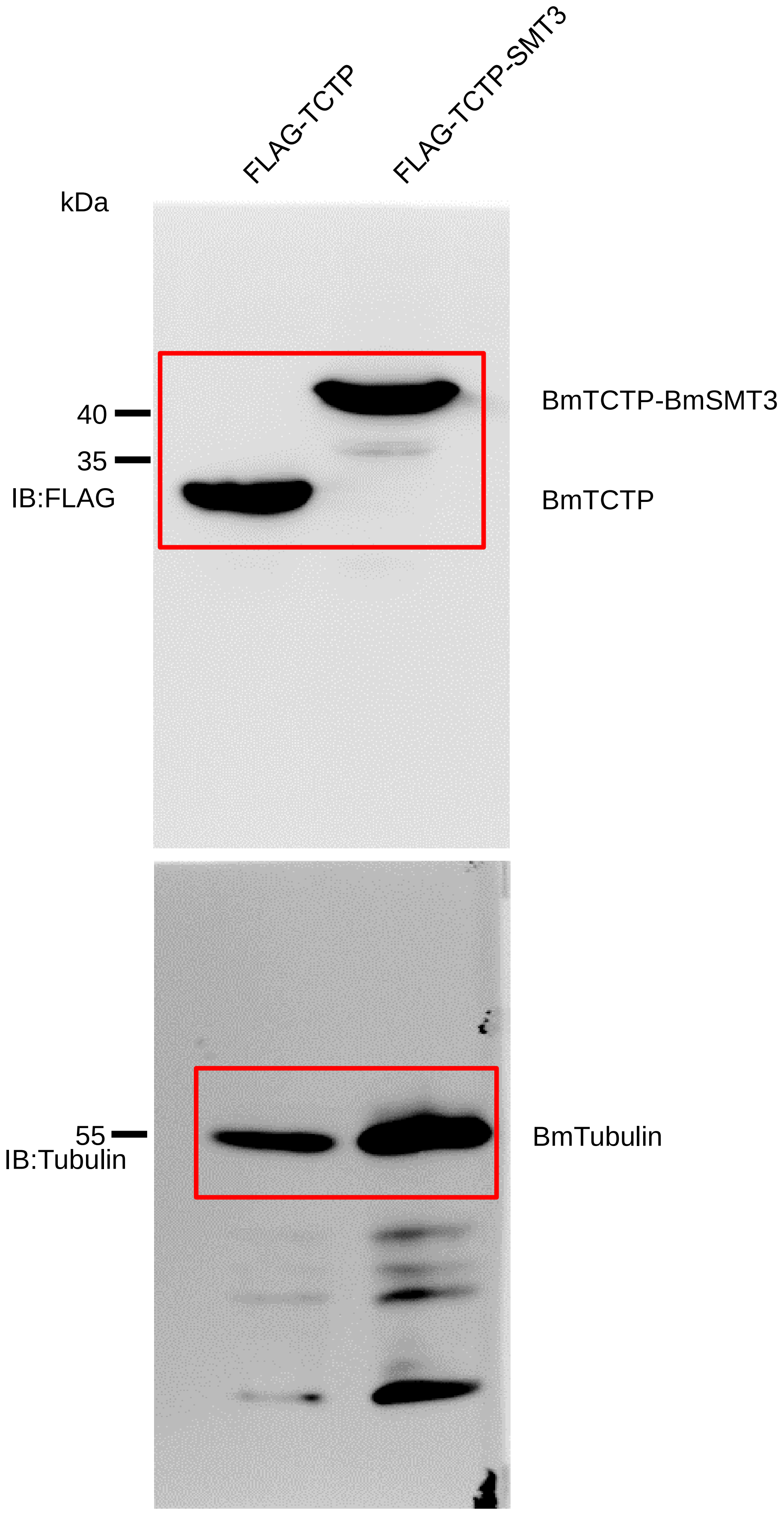

Related to Figure 4B

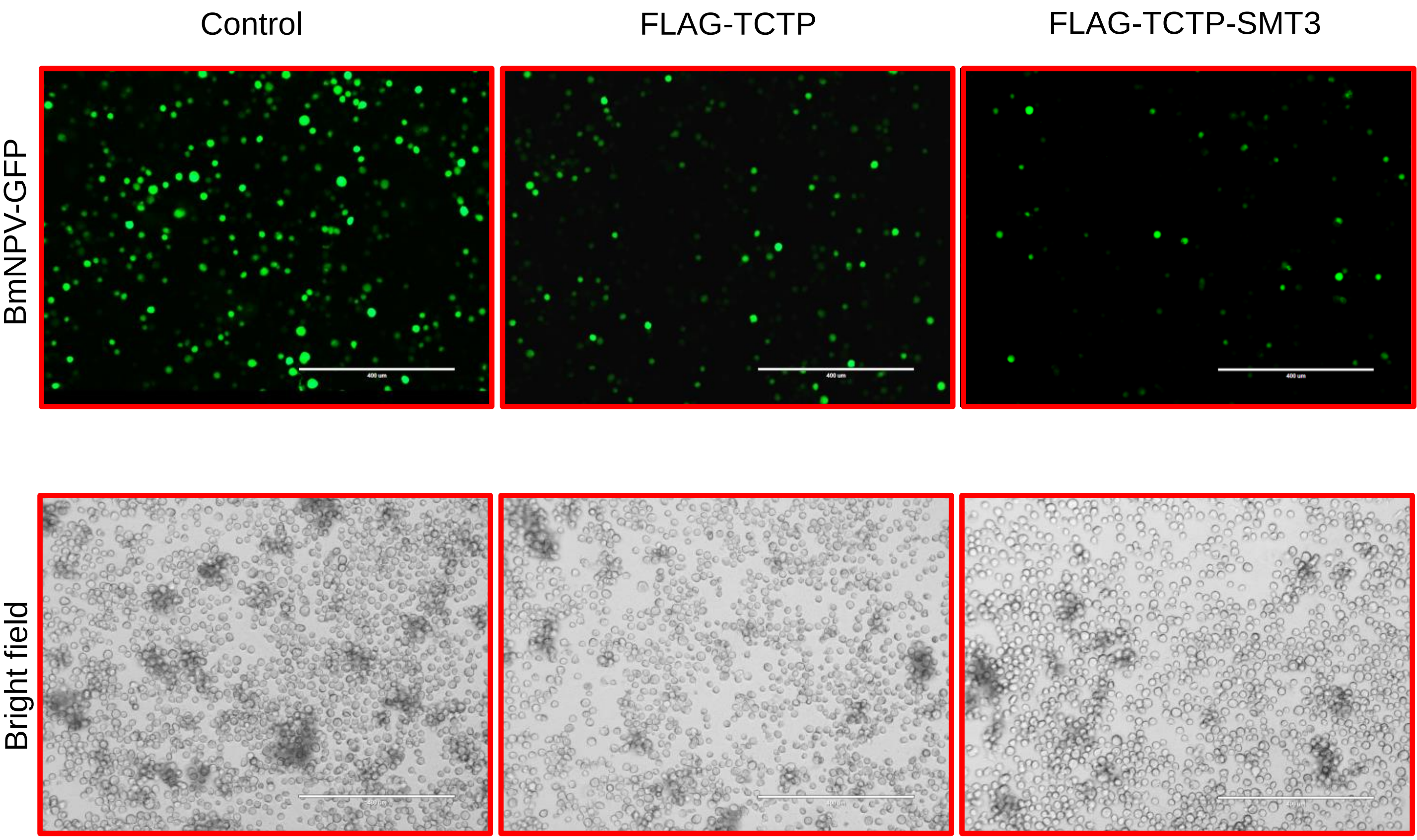

Related to Figure 4D

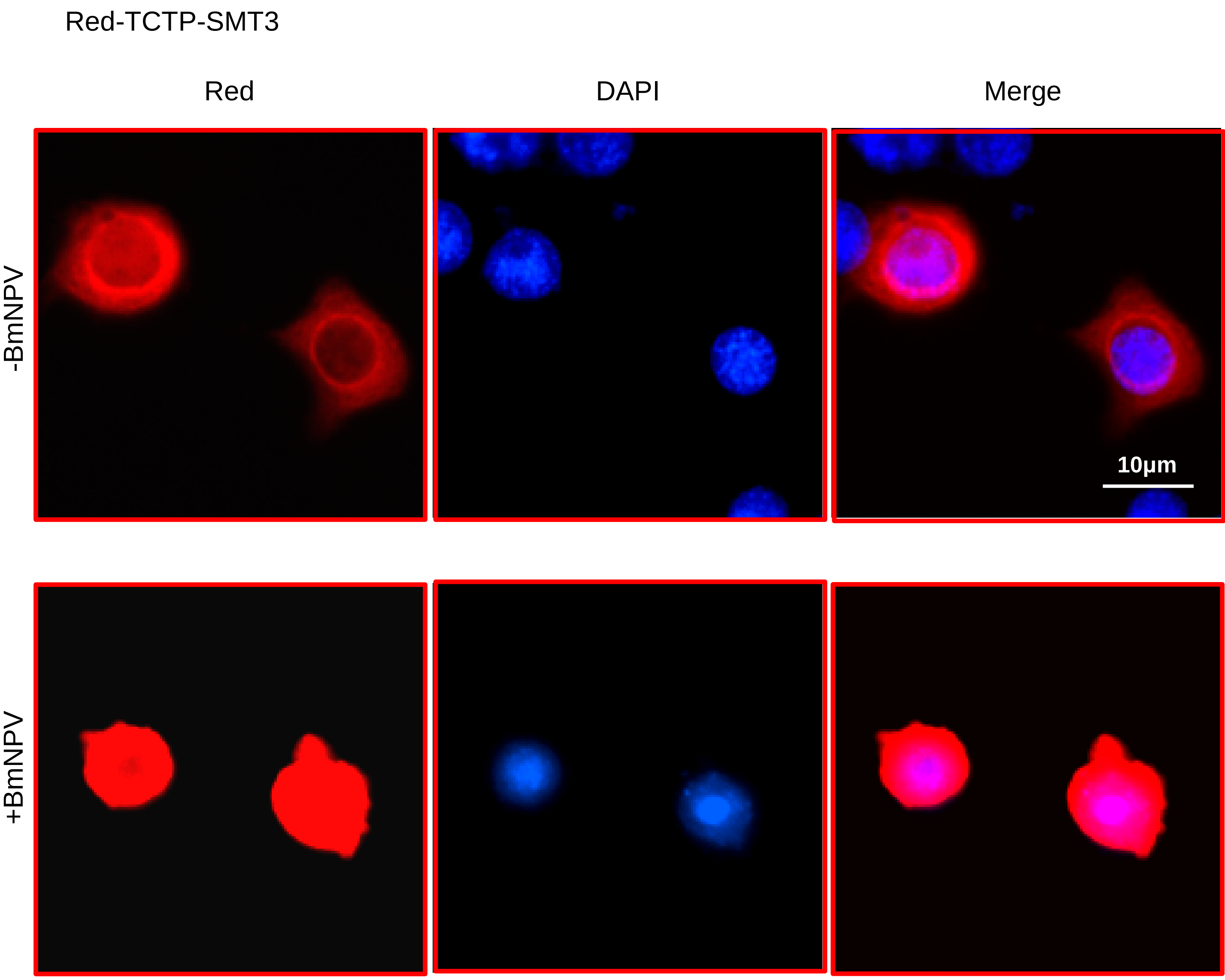

Related to Figure 5D

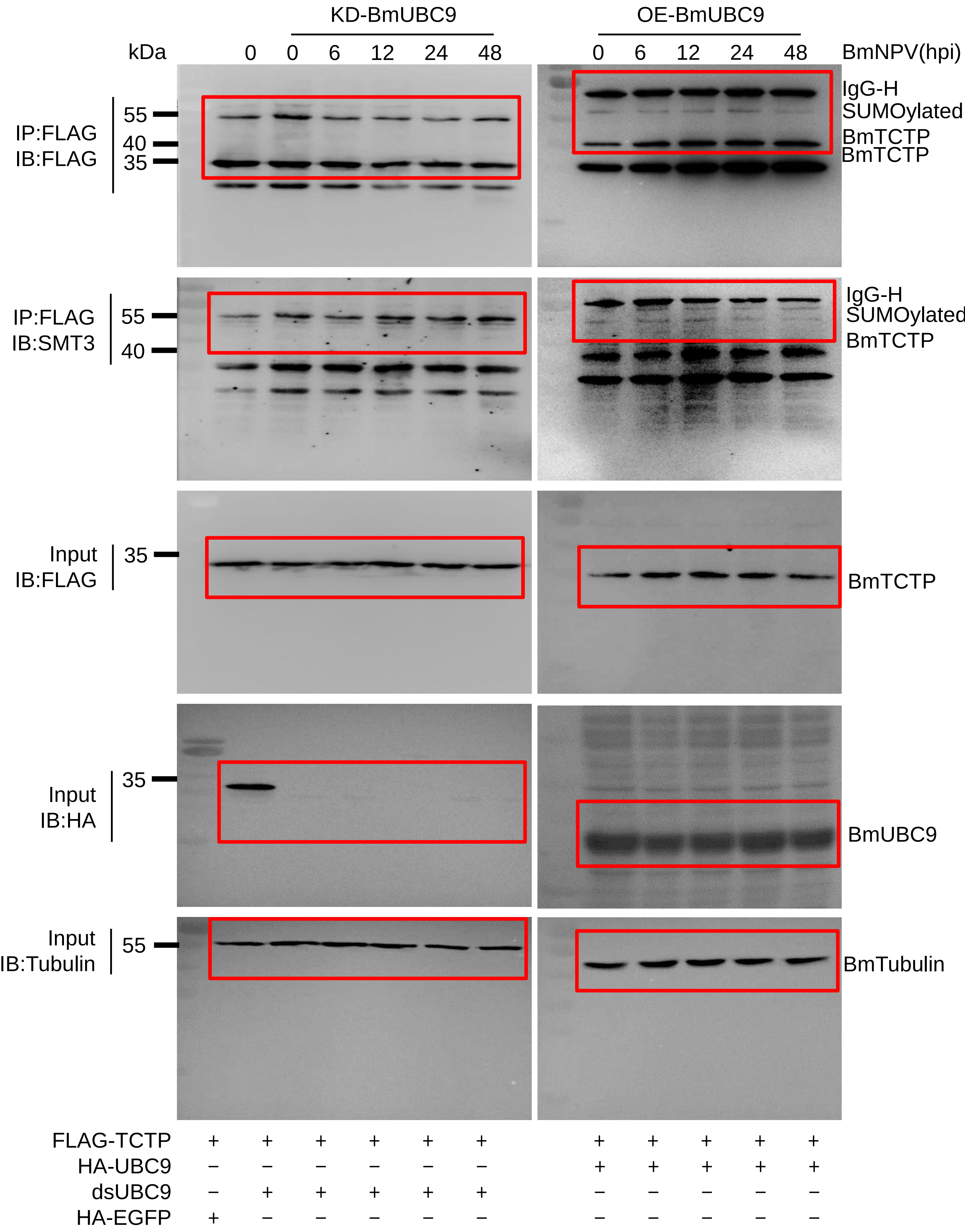

Related to Figure 6A

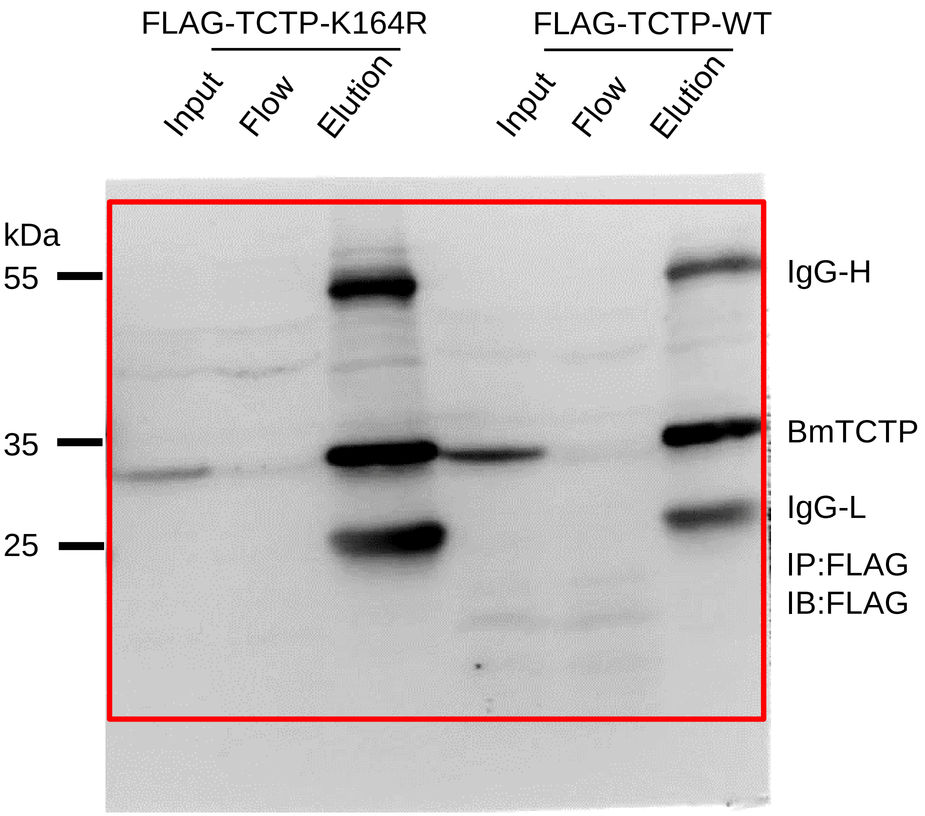

Related to Figure 7A

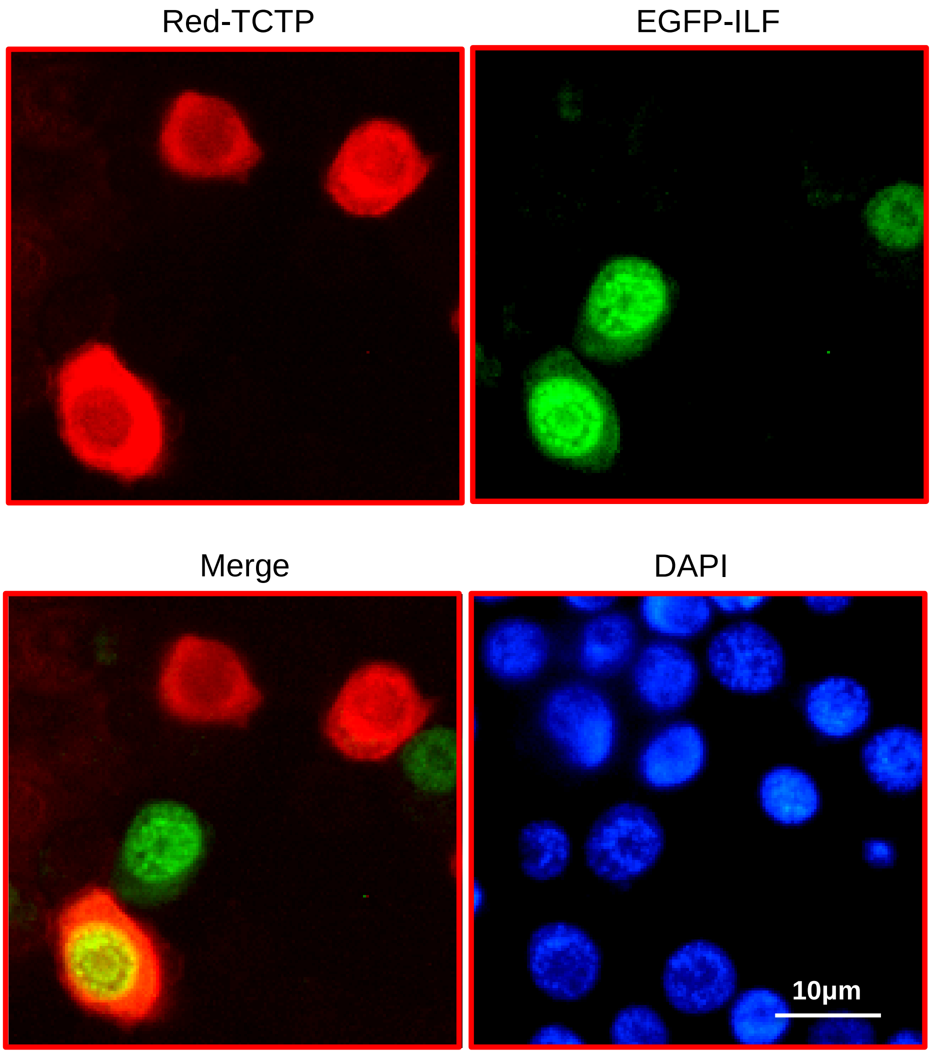

Related to Figure 7B

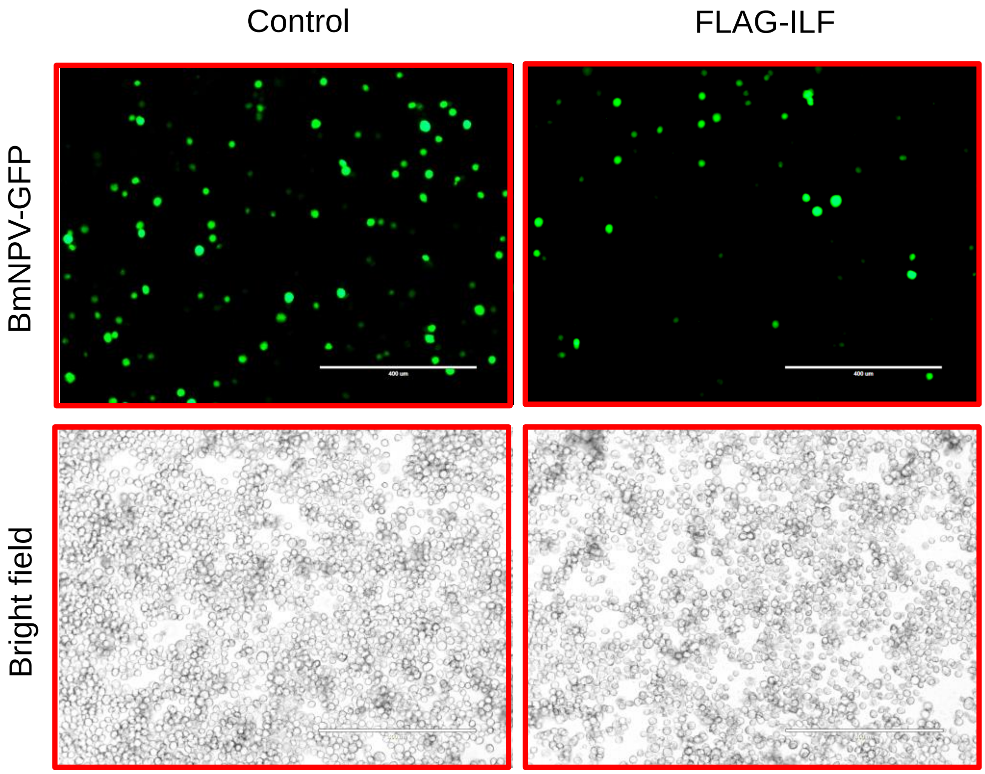

Related to Figure S1

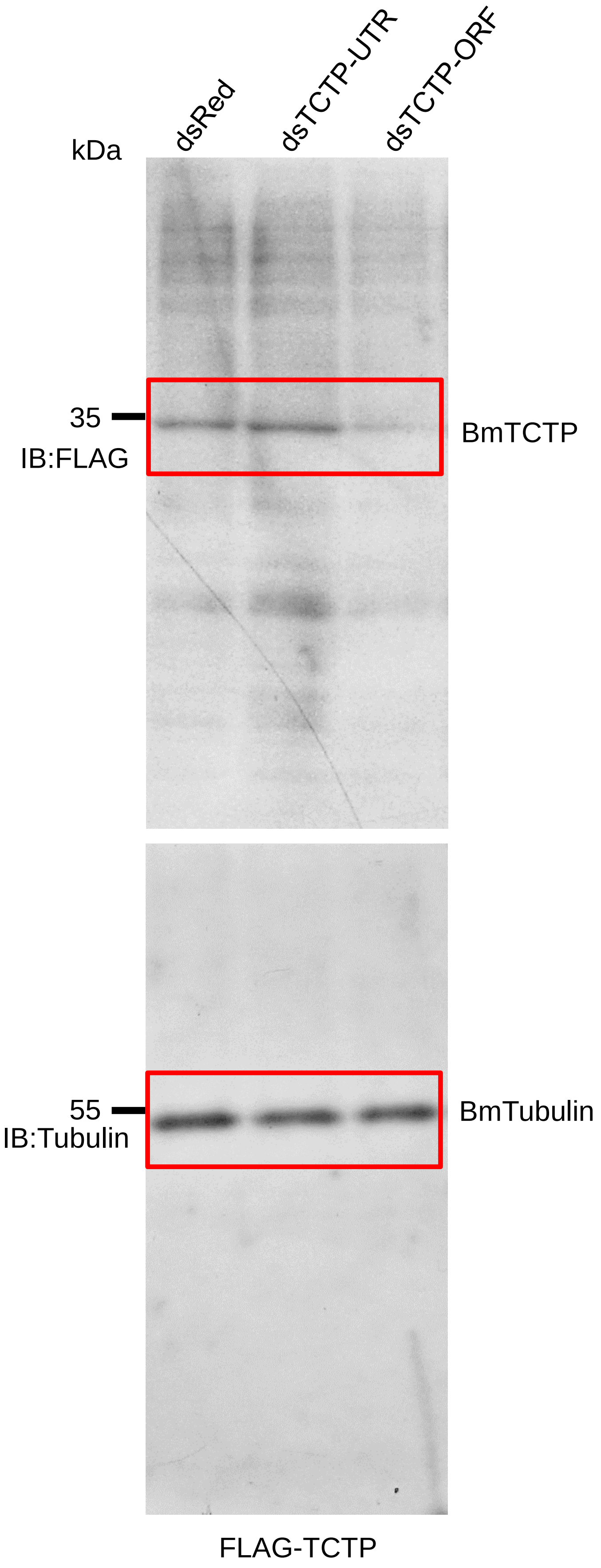

Supplement: Supplementary file 2 [file DataSheet_2.pdf]
